# The macromolecular complexes of histones affect protein arginine methyltransferase activities

**DOI:** 10.1016/j.jbc.2021.101123

**Published:** 2021-09-06

**Authors:** Melody D. Fulton, Mengtong Cao, Meng-Chiao Ho, Xinyang Zhao, Y. George Zheng

**Affiliations:** 1 Department of Pharmaceutical and Biomedical Sciences, College of Pharmacy, The University of Georgia, Athens, Georgia, USA; 2 Institute of Biological Chemistry, Academia Sinica, Nankang, Taipei, Taiwan; 3 Department of Biochemistry and Molecular Genetics, The University of Alabama at Birmingham, Birmingham, Alabama, USA

**Keywords:** PRMT, arginine methylation, histone, octamer, nucleosome, epigenetics, [^14^C]SAM, SAM-(methyl-^14^C), ADMA, asymmetric *N*^*G*^, *N*^*G*^-dimethylarginine, CARM1, coactivator-associated arginine methyltransferase 1, MMA, *N*^*G*^-monomethylarginine, PDB, Protein Data Bank, PRMT, protein arginine methyltransferase, PTM, post-translational modification, SDMA, symmetric *N*^*G*^, *N*’^*G*^-dimethylarginine

## Abstract

Histone arginine methylation is a key post-translational modification that mediates epigenetic events that activate or repress gene transcription. Protein arginine methyltransferases (PRMTs) are the driving force for the process of arginine methylation, and the core histone proteins have been shown to be substrates for most PRMT family members. However, previous reports of the enzymatic activities of PRMTs on histones in the context of nucleosomes seem contradictory. Moreover, what governs nucleosomal substrate recognition of different PRMT members is not understood. We sought to address this key biological question by examining how different macromolecular contexts where the core histones reside may regulate arginine methylation catalyzed by individual PRMT members (*i.e.*, PRMT1, PRMT3, PRMT4, PRMT5, PRMT6, PRMT7, and PRMT8). Our results demonstrated that the substrate context exhibits a huge impact on the histone arginine methylation activity of PRMTs. Although all the tested PRMTs methylate multiple free histones individually, they show a preference for one particular histone substrate in the context of the histone octamer. We found that PRMT1, PRMT3, PRMT5, PRMT6, PRMT7, and PRMT8 preferentially methylate histone H4, whereas PRMT4/coactivator-associated arginine methyltransferase 1 prefers histone H3. Importantly, neither reconstituted nor cell-extracted mononucleosomes could be methylated by any PRMTs tested. Structural analysis suggested that the electrostatic interaction may play a mechanistic role in priming the substrates for methylation by PRMT enzymes. Taken together, this work expands our knowledge on the molecular mechanisms of PRMT substrate recognition and has important implications for understanding cellular dynamics and kinetics of histone arginine methylation in regulating gene transcription and other chromatin-templated processes.

Over the past few decades, there has been significant strides in understanding the intricate mechanisms that regulate chromatin structure and how structural changes in the chromatin can enable or hinder gene transcription (1). The basic unit of the chromatin is the nucleosome, which consists of 146 base pairs of DNA wrapped around a protein core called the histone octamer (2). The histone octamer includes two copies of the low–molecular-weight proteins (11–14 kDa) and highly basic histone proteins: H4, H3, H2A, and H2B (3, 4). When the histones are not in the complex form of the nucleosome, the histones H3 and H4 form a stable tetramer, whereas H2A and H2B form a stable dimer (2). One of the mechanisms that can alter chromatin structure and impact gene transcription is the post-translational modifications (PTMs) on the nucleosomal core histones (5, 6). Among histone PTMs, multiple characteristic arginine methylation sites have been identified and correlate with different gene expression states (7, 8). Histone arginine methylation is introduced by protein arginine methyltransferases (PRMTs), which catalyze the formation of three major types of arginine methylation marks: *N*^*G*^-monomethylarginine (MMA), asymmetric *N*^*G*^, *N*^*G*^-dimethylarginine (ADMA), and symmetric *N*^*G*^, *N’*^*G*^-dimethylarginine (SDMA). PRMTs are classified based on the type of methyl marks they deposit: PRMT1, PRMT2, PRMT3, PRMT4 (coactivator-associated arginine methyltransferase 1 [CARM1]), PRMT6, and PRMT8 are type I enzymes generating MMA and ADMA; PRMT5 and PRMT9 are type II enzymes generating MMA and SDMA; and PRMT7 is a type III enzyme generating only MMA (9).

Nucleosomal histones are important cellular substrates of PRMTs, and histone arginine methylation plays important roles in regulating chromatin dynamics, transcriptional activation or repression, and DNA damage repair (6, 8, 10, 11, 12, 13, 14). Individual PRMT enzymes exhibit varied site specificity on the histone substrates. PRMT1 asymmetrically dimethylates histone H4 at the arginine-3 site (H4R3) in mammalian cells, and this event correlates with transcriptional activation in gene expression regulation (15, 16, 17, 18, 19). PRMT8, the closest homolog of PRMT1, methylates free histone H4 protein (20). Overexpressed PRMT3 in human embryonic kidney 293 cells methylates H4 and increases H4R3me2a level in cells (21). In agreement, recombinant PRMT3 methylates histone H4 peptides *in vitro* (22, 23). PRMT4 (CARM1) methylates histone H3 at multiple sites: R2, R17, and R26, and promotes gene activation (24, 25). H3R17/26 methylation is critical to early mouse embryo development and paternal genome reprogramming (26, 27).

PRMT5 is reported to methylate multiple histone proteins H2A, H3, and H4. PRMT5 can methylate histones H2A and H4 to generate H2AR3me2s and H4R3me2s modifications that are associated with gene repression (28, 29, 30). PRMT5 is highly expressed in embryonic stem cells and selectively methylates cytosolic H2AR3 (31). Furthermore, the H3R8 site is symmetrically dimethylated by the SWItch/Sucrose nonfermentable–associated PRMT5, and this methylation is related to H4R3me2s methylation and leads to transcriptional repression (32, 33). The H3R8 site can also be subjected to asymmetric dimethylation by PRMT2, and the H3R8me2a methylation mark at promoters and enhancers is required for the maintenance of target gene expression (34).

H3R2 is asymmetrically dimethylated by PRMT6, forming H3R2me2a, which antagonizes the mixed lineage leukemia 1 complex from methylating H3K4 (35, 36, 37). Therefore, H3R2me2a inhibits H3K4 trimethylation and acts as a repressive mark, and PRMT6 is associated with transcriptional repression of tumor suppressor genes (38, 39). In contrast, PRMT5-caused symmetric dimethylation of H3R2 (H3R2me2s) is associated with gene activation (40). Mechanistically, H3R2me2s is permissive for binding with WD-40 repeat–containing protein 5, whereas H3R2me2a is preventive (37, 41). H3R42 is another site methylated by both CARM1 and PRMT6, and the methylation stimulates transcription (42). On histone H2A, except the R3 site, R11 and R29 can also be methylated by PRMT1 and PRMT6, and notably, H2AR29me2 is specifically enriched at genes repressed by PRMT6, implicating the role of H2AR29me2 in transcriptional repression (43).

The pseudodimeric enzyme PRMT7 is only capable of catalyzing the formation of monomethylarginine (*i.e.*, type III activity). PRMT7 methylates all the four core histones in the free protein form: H2A, H2B, H3, and H4 (44). In particular, PRMT7 specifically recognizes arginine residues within an RXR motif in arginine- and lysine-rich regions, such as H2BR29, H2BR31, and H4R17 (45, 46). PRMT7-mediated monomethylation of H4R17 allosterically potentiates PRMT5 activity on H4R3 (47). PRMT9 does not have methyltransferase activity on the nuclear histones (48). There are additional arginine methylation sites in histones, but their biochemical and functional properties remain to be characterized (13).

While many PRMTs are reported to methylate histones, and in some cases also the nucleosome (*e.g.*, PRMT5 (30) and PRMT1 (16)), there has yet to be a systematic study that comparatively examines how the histone assemblies affect PRMT substrate specificity among individual PRMT family members. Such information is particularly important for assessing how the substrate interaction and the methylation activity of PRMTs are altered by the context of substrates. For instance, several studies have demonstrated that different PTM patterns in the histone substrates exhibit sophisticated influence on the activities of PRMTs in arginine methylation (47, 49, 50, 51, 52, 53). To better mechanistically understand how different PRMT enzymes access and methylate cellular histone proteins for epigenetic regulation, in this work, we performed detailed methylation studies of histone arginine methylation under different macromolecular contexts: the individual-free histones, histone octamers, and mononucleosomes (Fig. 1), catalyzed by different human PRMT proteins: PRMT1, PRMT3, PRMT4, PRMT5, PRMT6, PRMT7, and PRMT8. PRMT2 and PRMT9 were not included in this study because the recombinantly expressed PRMT2 is inactive in histone methylation assays (42, 54), and PRMT9 is a nonhistone methyltransferase (48). Also, given that H3.3 is incorporated at loci of active gene transcription and its expression is not limited to a specific phase in the cell cycle (unlike H3.1 and H3.2) (55), we used the histone octamer and mononucleosomes containing recombinant full-length H3.3 in these experiments. Our data found that different macromolecular contexts drastically impacted the activities of all the PRMTs examined.

**Figure 1:**
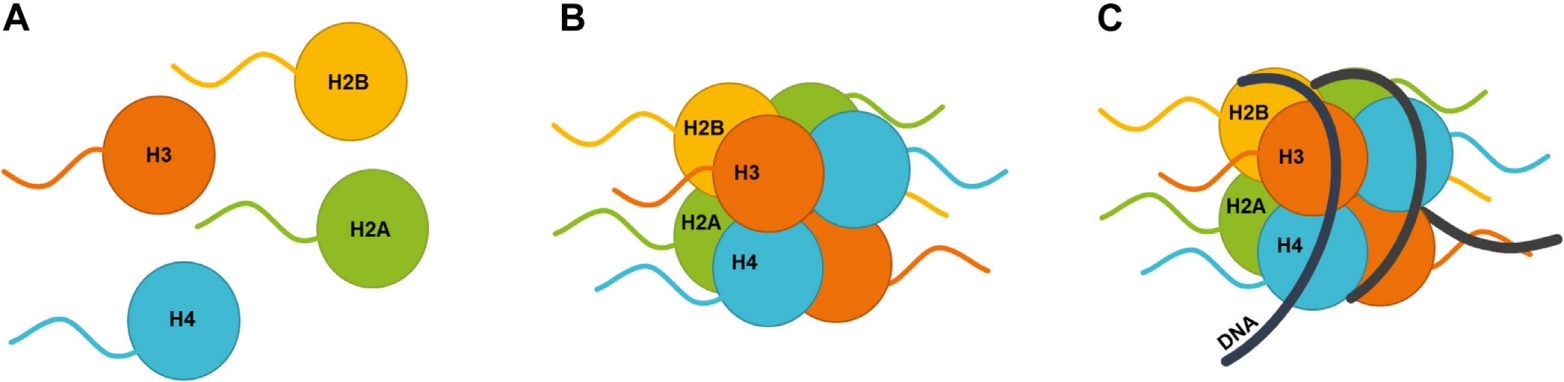
**The three macromolecular contexts of histone substrates tested in this study.***A*, free histones, *B*, histone octamers, and *C*, nucleic acid–bound octamers (*i.e.*, nucleosomes).

## Results

### PRMT1 methylates histones H2A and H4 individually yet favors methylation of histone H4 in the context of the histone octamer

We used a gel-based phosphorimaging methyltransferase assay to determine the substrate specificity of different PRMTs in histone methylation (52). The reaction solutions contained individual PRMT enzyme, SAM-(methyl-^14^C) [^14^C]SAM, and methyl acceptor substrates such as individual recombinant human histones (H2A, H2B, H3.3, and H4), histone octamer, mononucleosome, or a peptide with a sequence of the N-terminal tail of histone H4, H3, or H2A. Given that two copies of each histone exist in the histone octamer, we calculated the appropriate amount of histone octamer to achieve equal molar concentrations of histone in each reaction. Methylation reactions proceeded for 30 min (unless noted otherwise), quenched with 6× Tris–tricine gel loading buffer, and heated for 10 min at 95 °C. Proteins were resolved by 16% SDS-PAGE using a Tris–tricine buffer system, and then stained with Coomassie brilliant blue to visualize protein loading across lanes. Afterward, gels were dried for 2 h at 70 °C and then exposed to a storage phosphor screen (PerkinElmer) for 72 h in the dark, unless stated otherwise.

**Figure 2:**
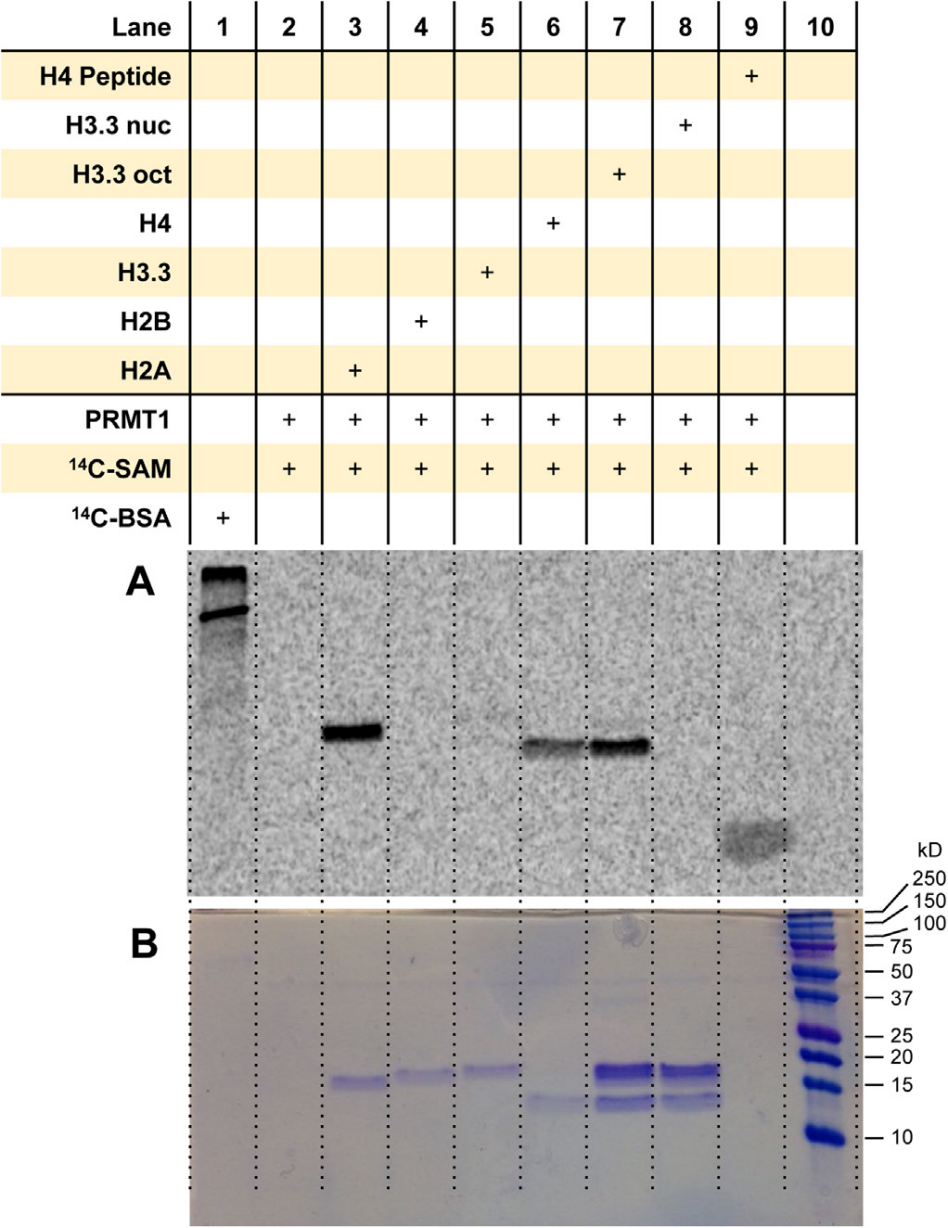
**Arginine methylation of the histones in different contexts by PRMT1.** Reactions were held for 30 min at 30 °C with 0.05 μM PRMT1, 5 μM [^14^C]SAM, and 1 μM of substrate. Samples were resolved by SDS-PAGE with a 16% polyacrylamide gel, stained with Coomassie blue, and then dried for 2 h. Dried gels were exposed to the phosphor screen for 72 h in the dark. [^14^C]BSA and a protein ladder were used in lanes 1 and 10 as a radiolabeled protein marker and a protein MW reference, respectively. *A*, phosphor image of radiolabeled proteins. *B*, Coomassie blue protein staining of the respective samples from the same gel. [^14^C]BSA, [^14^C]-labeled bovine serum albumin; [^14^C]SAM, SAM-(methyl-^14^C); MW, molecular weight; PRMT1, protein arginine methyltransferase 1.

It is well known that PRMT1 asymmetrically dimethylates histone H4 at the arginine-3 site (H4R3), and this event promotes transcriptional activation (15, 16, 56, 57, 58). Also, PRMT1 has been reported to methylate oligonucleosomes extracted from 6C2 cells, which was regarded as an essential step to promote histone acetylation (16). We sought to understand how PRMT1 may behave differently when the histone substrates are presented in varying forms: short peptide (Ac-H4(1–20)), individual full-length recombinant histones, recombinant histone octamer, and recombinant mononucleosome. As shown in Figure 2 and Fig. S1, PRMT1 heavily methylated H2A and H4 but did not methylate H2B or H3.3 in the full-length protein form. We did not observe arginine methylation of the recombinant mononucleosomes by PRMT1, which is consistent with a previous report that used recombinant mononucleosomes (52). Under these reaction conditions, we clearly observed arginine methylation of the Ac-H4(1–20) peptide (Fig. 2, *lane 9*). To further understand the pattern of substrate specificity of PRMT1 on histones, we performed the methyltransferase assay with the H2A/H2B dimer, H3/H4 tetramer, histone H3.1 octamer, and histone H3.3 octamer. As seen in Fig. S1, *E*–*H*, in the context of H2A/H2B dimer, PRMT1 strongly methylated H2A, which was similar to the methylation of free H2A (comparing lane 3 and lane 8). However, when the H3/H4 tetramer was added to the H2A/H2B dimer solution, H2A could not be methylated anymore and instead PRMT1 solely methylated H4 (lane 5). Therefore, the H2A methylation site is shielded by the H3/H4 tetramer, and only the H4 methylation site is accessible by PRMT1 in the octamer state, which explains why H2A is only methylated in the free H2A and the H2A/H2B dimer forms but not in the octamers. Overall, PRMT1 is capable of methylating the individual histones H2A and H4 proteins but exhibited a preference for histone H4 in the context of the histone octamer.

### PRMT3 methylates histones H4 and H2A individually, and the N-terminal region of PRMT3 is important for methylation of the histone octamer

**Figure 3:**
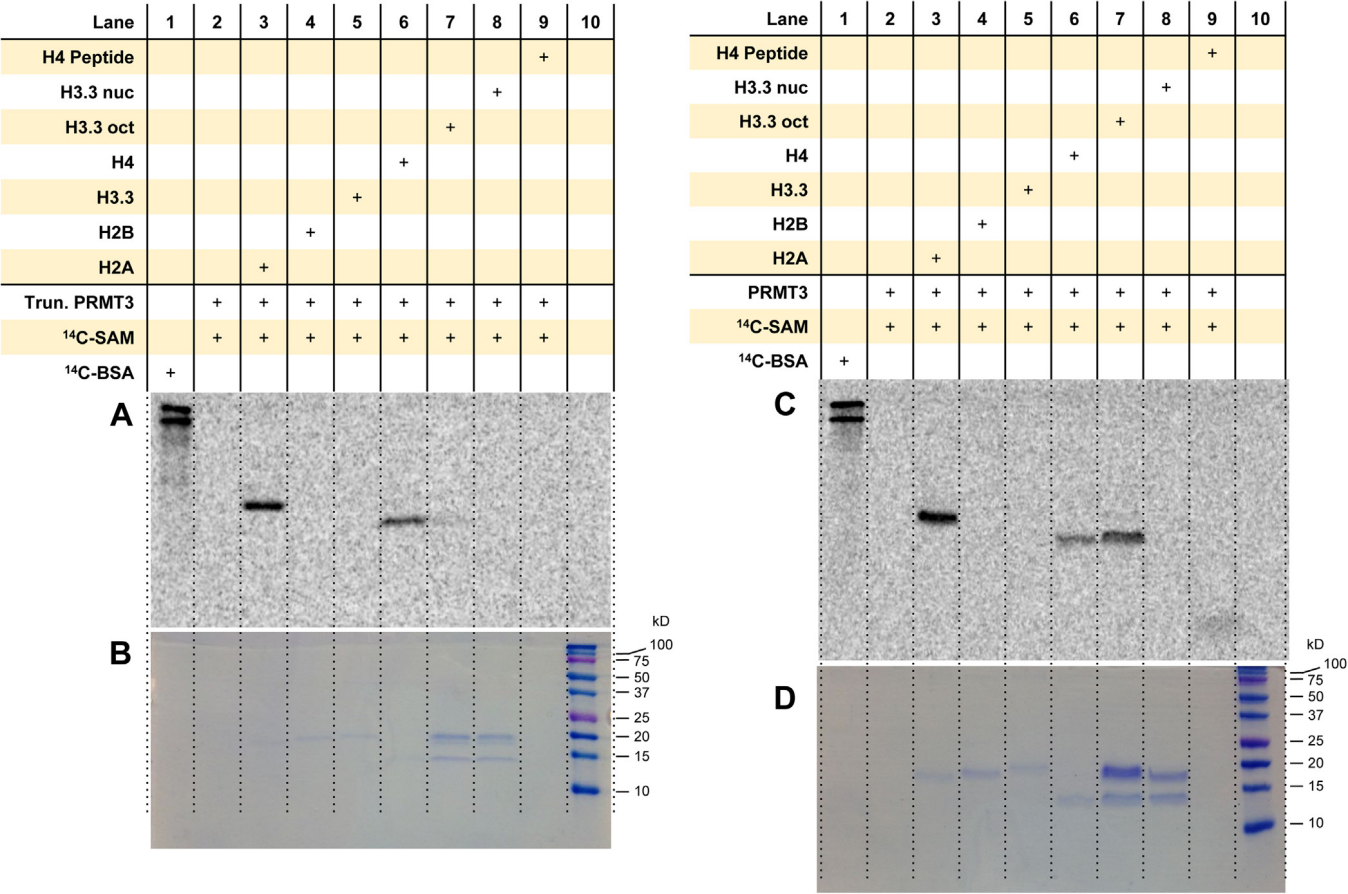
**Arginine methylation of histones and mononucleosomes by PRMT3.** Reactions were held for 30 min at 30 °C with 0.05 μM truncated (*A* and *B*) or full-length PRMT3 (*C* and *D*), 5 μM [^14^C]SAM, and 1 μM of substrate. Samples were resolved by SDS-PAGE with a 16% polyacrylamide gel, stained with Coomassie blue, and then dried for 2 h. Dried gels were exposed to the phosphor screen for 96 h in the dark. [^14^C]BSA and a protein ladder were used in lanes 1 and 10 as a radiolabeled protein marker and a protein MW reference, respectively. *A* and *C*, phosphor images of radiolabeled proteins. *B* and *D*, Coomassie blue protein staining of the respective samples from the same gel. [^14^C]BSA, [^14^C]-labeled bovine serum albumin; [^14^C]SAM, SAM-(methyl-^14^C); MW, molecular weight; PRMT3, protein arginine methyltransferase 3.

Thus far, there are a few reports that mention the use of histone peptides (only H4-based sequences) as a substrate of PRMT3 (22, 23, 49, 59), and there is one report that examines the specific histones that PRMT3 methylates when provided the histone octamer (42). Also, the N-terminal region of rat PRMT3 has been reported to be important for substrate specificity (60). To determine whether the N-terminal region of human PRMT3 influences substrate preference with histones, we performed the assay with both truncated (residues 211–531, Protein Data Bank [PDB] entry: 2FYT) and full-length (residues 1–544) PRMT3 (Fig. 3, Figs. S2 and S3). In the recombinant-free protein forms, the truncated PRMT3 strongly methylated histone H2A in comparison to H4 but did not appear to methylate H2B or H3 (Fig. 3, *A* and *B*). When the substrate was the histone octamer, there was only a weak detection of arginine methylation on H4 (Fig. 3*A*, *lane 7*). The full-length PRMT3 strongly methylated the individual histone H2A over H4, whereas H2B and H3.3 were not methylated (Fig. 3, *C* and *D*). Unlike the truncated PRMT3, the full-length PRMT3 clearly methylated the histone octamer and preferred histone H4 in this macromolecular context (Fig. 3*C*, *lane 7*). Also, strong arginine methylation of the Ac-H4(1–20) peptide was observed with only the full-length PRMT3 under these reaction conditions. Regardless of truncated or full-length PRMT3, in the context of nucleosome, no histones were observed to be methylated (lane 8 in Fig. 3, *A* and *C*). These results demonstrate that PRMT3 methylates histones H2A and H4 in the free protein form but only methylates H4 in the octamer form, and the N-terminal domain of PRMT3 impacts histone arginine methylation.

### PRMT4/CARM1 methylates all the core histones, yet prefers to methylate H3.3 in the context of the histone octamer

**Figure 4:**
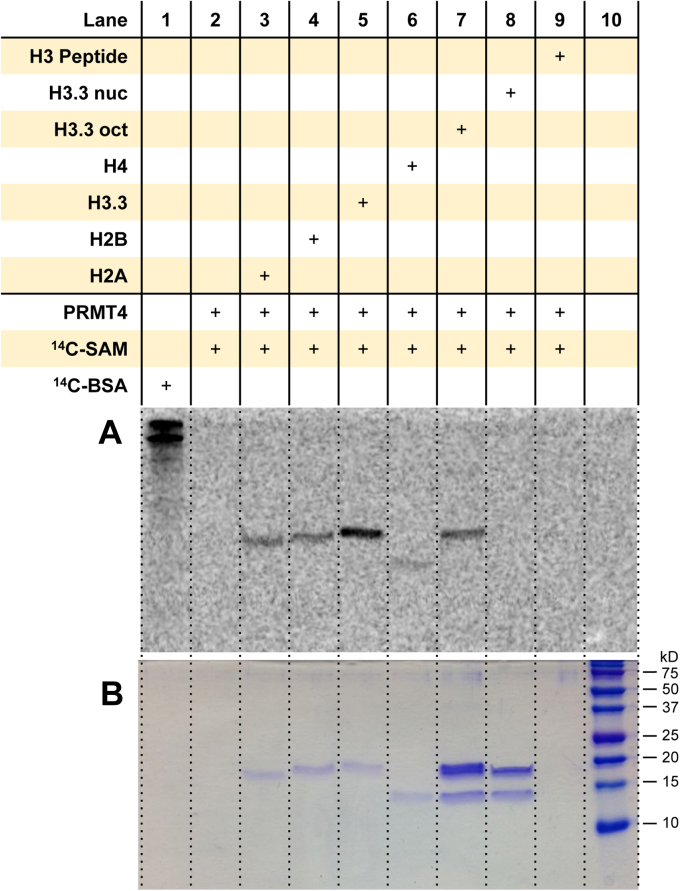
**Arginine methylation of histones and mononucleosomes by PRMT4.** Reactions were held for 1 h at 30 °C with 0.05 μM PRMT4, 5 μM [^14^C]SAM, and 1 μM of substrate. Samples were resolved by SDS-PAGE with a 16% polyacrylamide gel, stained with Coomassie blue, and then dried for 2 h. Dried gels were exposed to the phosphor screen for 96 h in the dark. [^14^C]BSA and a protein ladder were used in lanes 1 and 10 as a radiolabeled protein marker and a protein MW reference, respectively. *A*, phosphor image of radiolabeled proteins. *B*, Coomassie blue protein staining of the respective samples from the same gel. [^14^C]BSA, [^14^C]-labeled bovine serum albumin; [^14^C]SAM, SAM-(methyl-^14^C); MW, molecular weight; PRMT4, protein arginine methyltransferase 4.

To determine the substrate specificity of PRMT4/CARM1 with the various histone substrates, we performed radioactive methylation assays based on the reaction conditions used by Zhang *et al.* (61). To improve the signal-to-background noise, the exposure time was extended to 96 h for the dried gel with the phosphor screen. With these conditions, PRMT4 methylates all the recombinant histones individually, and the order of labeling intensity appeared to be H4 < H2A, H2B < H3.3, with H3.3 being the most heavily methylated substrate (Fig. 4 and Fig. S4). In the context of the histone octamer, PRMT4 strongly methylated H3.3 but not any other histones. The substrate preference toward H3 by PRMT4 was in agreement with previous studies showing that PRMT4 methylates multiple sites in H3 (24, 25, 62). Similar to PRMT1 and PRMT3, we did not observe any arginine methylation of the mononucleosomes by PRMT4, in agreement with a previous report (24). Also, methylation of the Ac-H3(1–20) peptide was not observable under these reaction conditions, likely because the methylation site R-17 is too close to the carboxyl end as the C-terminal residues were previously shown to be important for substrate recognition by PRMT4 (63). Together, the data demonstrate that PRMT4 methylates individual histones H2A, H2B, H3.3, and H4 in the recombinant protein form and yet shows a preference for methylating histone H3.3 alone in the macromolecular context of octamers.

### PRMT5 and PRMT5/MEP50 methylate histones H2A and H4, yet strongly methylate H4 alone in the context of the histone octamer

**Figure 5:**
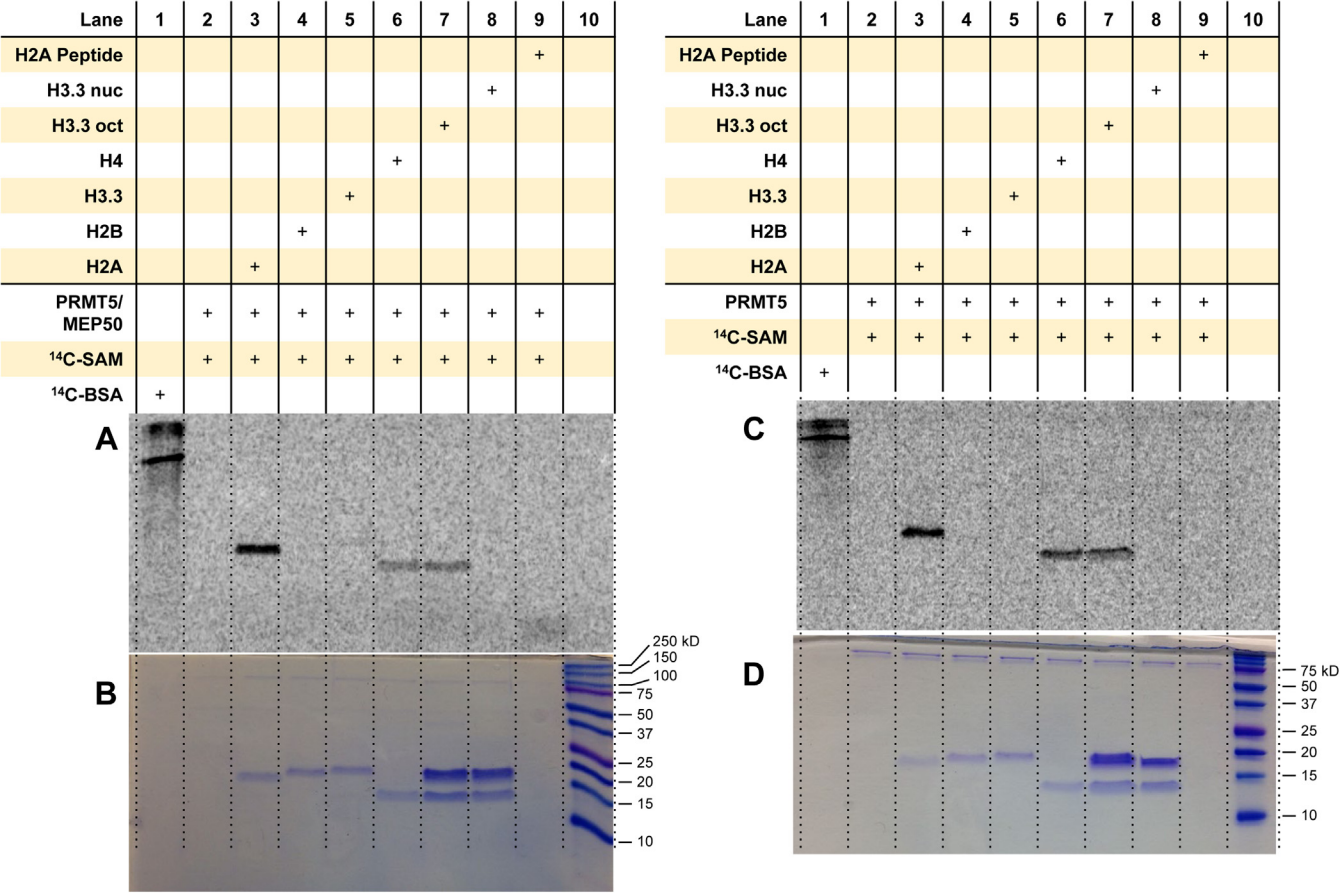
**Arginine methylation of histones and mononucleosomes by PRMT5.** Reactions were held for 1 h at 30 °C with 0.05 μM PRMT5/MEP50 (*A* and *B*) or 0.2 μM PRMT5 (*C* and *D*), 5 μM [^14^C]SAM, and 1 μM of substrate. Samples were resolved by SDS-PAGE with a 16% polyacrylamide gel, stained with Coomassie blue, and then dried for 2 h. Dried gels with PRMT5/MEP50 and PRMT5 samples were exposed to the phosphor screen for 72 and 96 h in the dark, respectively. [^14^C]BSA and a protein ladder were used in lanes 1 and 10 as a radiolabeled protein marker and a protein MW reference, respectively. *A* and *C*, phosphor images of radiolabeled proteins from the reactions with PRMT5/MEP50 and PRMT5, respectively. *B* and *D*, Coomassie blue protein–stained gels for PRMT5/MEP50 and PRMT5, respectively. [^14^C]BSA, [^14^C]-labeled bovine serum albumin; [^14^C]SAM, SAM-(methyl-^14^C); MW, molecular weight; PRMT5, protein arginine methyltransferase 5.

We performed the phosphorimaging assay to determine whether there were differences in the substrate specificity of PRMT5 alone *versus* PRMT5–MEP50 complex when given individual histones, octamers, or mononucleosomes (Figs. 5, Figs. S5 and S6). Both PRMT5 and PRMT5/MEP50 strongly methylated recombinant H2A and H4 (Fig. 5). Interestingly, PRMT5 methylated H2A and H4 approximately at an equivalent level, but PRMT5/MEP50 showed a higher methylation activity on H2A in comparison to H4. Neither PRMT5 nor PRMT5/MEP50 could methylate H2B. In the context of the histone octamer, PRMT5 and PRMT5/MEP50 only methylated H4, and arginine methylation on the other core histones was absent. We observed greater arginine methylation of the Ac-H2A(1–21) peptide and H3.3 (faint band in Fig. 5*A*, *lane 5* and *lane 9*) by PRMT5/MEP50 than PRMT5 alone, which affirms that MEP50 plays a role in modulating substrate specificity of PRMT5 (51, 64, 65). Also, PRMT5 and PRMT5/MEP50 did not methylate the mononucleosome (*lane 8*, Fig. 5, *A* and *C*).

### PRMT6 methylates the individual histones H2A, H3.3, and H4, yet strongly methylates H4 in the histone octamer

**Figure 6:**
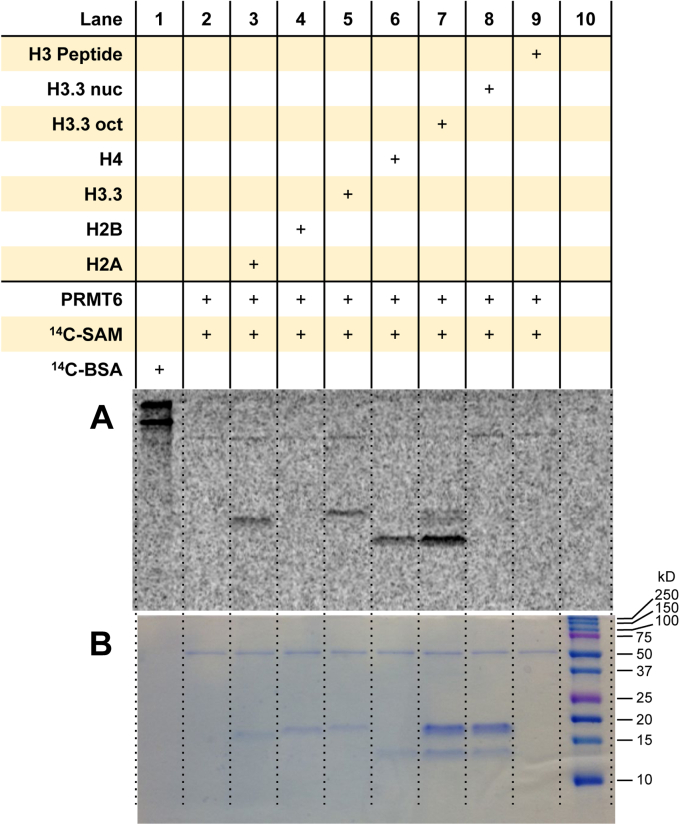
**Arginine methylation of histones and mononucleosomes by PRMT6.***A*, reactions were held for 3 h at 30 °C with 0.5 μM PRMT6, 5 μM [^14^C]SAM, and 1 μM of substrate. Samples were resolved by SDS-PAGE with a 16% polyacrylamide gel, stained with Coomassie blue, and then dried for 2 h. Dried gels were exposed to the phosphor screen for 96 h in the dark. [^14^C]BSA and a protein ladder were used in lanes 1 and 10 as a radiolabeled protein marker and a protein MW reference, respectively. *A*, phosphor image of radiolabeled proteins. *B*, Coomassie blue protein staining of the respective samples from the same gel. [^14^C]BSA, [^14^C]-labeled bovine serum albumin; [^14^C]SAM, SAM-(methyl-^14^C); MW, molecular weight; PRMT6, protein arginine methyltransferase 6.

We initially performed the radioactive methyltransferase assay using the conditions used for PRMT1 (30 min at 30 °C with 0.05 μM enzyme, 5 μM [^14^C]SAM, and 1 μM of substrate); however, histone arginine methylation by PRMT6 was not observed (data not shown). To improve the signal-to-background noise, the incubation time for the dried gel with the phosphor screen was increased to 96 h. Also, the reaction time was increased to 3 h at 30 °C with 0.5 μM PRMT6, 5 μM [^14^C]SAM, and 1 μM of substrate. With these conditions, a faint higher molecular weight band was observed in nearly all the lanes with PRMT6, which was caused by PRMT6 automethylation (Fig. 6 and Fig. S7). PRMT6 methylated the individual histone proteins H2A, H3.3, and H4, with strongest activity on H4. In the context of the histone octamer, an even greater level of radiolabeled H4 was observed in comparison to H2A and H3.3. Consistent with the other PRMTs, PRMT6 does not methylate the mononucleosome. Also, arginine methylation of the Ac-H3(1–20) peptide was not detectable under these reaction conditions.

### PRMT7 methylates the histones H2A, H2B, and H4 individually, yet strongly methylates H4 alone in the histone octamer

**Figure 7:**
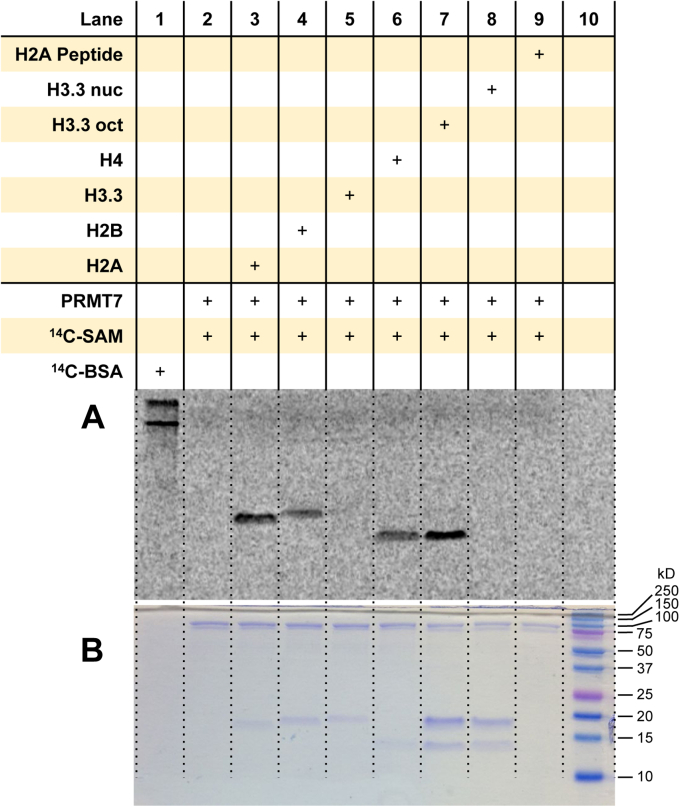
**Arginine methylation of histones and mononucleosomes by PRMT7.***A*, reactions were held for 3 h at 30 °C with 0.5 μM PRMT7, 5 μM [^14^C]SAM, and 1 μM of substrate. Samples were resolved by SDS-PAGE with a 16% polyacrylamide gel, stained with Coomassie blue, and then dried for 2 h. Dried gels were exposed to the phosphor screen for 96 h in the dark. [^14^C]BSA and a protein ladder were used in lanes 1 and 10 as a radiolabeled protein marker and a protein MW reference, respectively. *A*, phosphor image of radiolabeled proteins. *B*, Coomassie blue protein staining of the respective samples from the same gel. [^14^C]BSA, [^14^C]-labeled bovine serum albumin; [^14^C]SAM, SAM-(methyl-^14^C); MW, molecular weight; PRMT7, protein arginine methyltransferase 7.

As with PRMT6, we did not observe arginine methylation of the histones by PRMT7 under the reaction conditions that were applied to PRMT1 (data not shown). We applied the same exposure time and reaction conditions to PRMT7 that were used for PRMT6 (3-h reaction time and a 96-h exposure with the phosphor screen). We observed arginine methylation of the individual histones H2A, H2B, and H4 by PRMT7, albeit H2A was the most heavily methylated substrate (Fig. 7 and Fig. S8). Despite this observation, PRMT7 strongly methylated only histone H4 when presented with the histone octamer. Consistent with the other PRMTs examined, arginine methylation was not observed with the mononucleosome. Also, arginine methylation was not observed with H2A(1–21) under these conditions.

### PRMT8 methylates histones H2A, H3.3, and H4 individually, yet prefers to methylate H4 alone in the histone octamer

**Figure 8:**
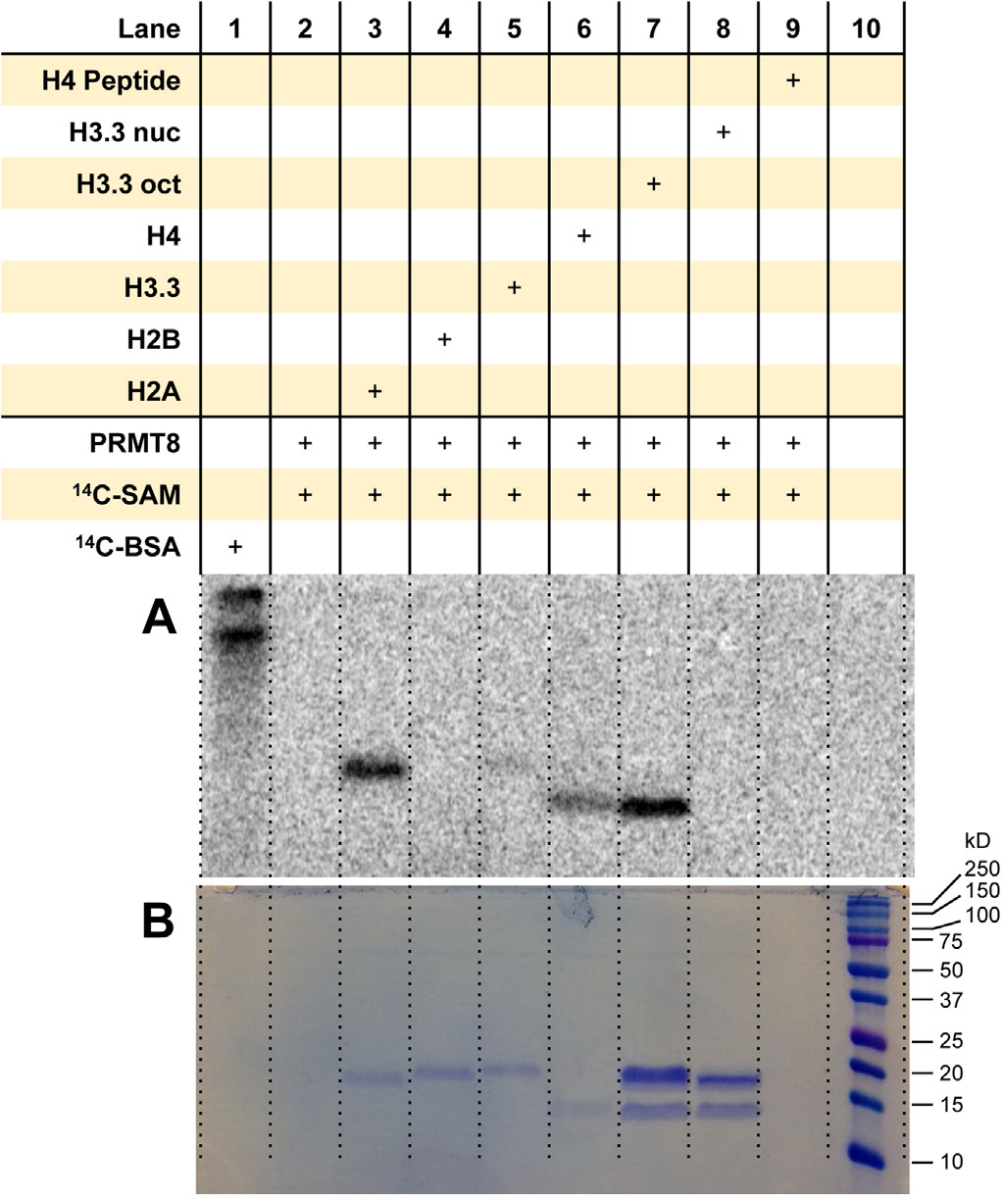
**Arginine methylation of histones and mononucleosomes by PRMT8.***A*, reactions were held for 30 min at 30 °C with 0.05 μM PRMT8, 5 μM [^14^C]SAM, and 1 μM of substrate. Dried gels were exposed to the phosphor screen for 72 h in the dark. [^14^C]BSA and a protein ladder were used in lanes 1 and 10 as a radiolabeled protein marker and a protein MW reference, respectively. *A*, phosphor image of radiolabeled proteins. *B*, Coomassie blue protein staining of the respective samples from the same gel. [^14^C]BSA, [^14^C]-labeled bovine serum albumin; [^14^C]SAM, SAM-(methyl-^14^C); MW, molecular weight; PRMT8, protein arginine methyltransferase 8.

Given PRMT8 shares the highest degree of structural homology with PRMT1 (66), one might expect PRMT8 and PRMT1 to methylate histones in a similar pattern. Interestingly, we observed a slight divergence of PRMT8 activity from PRMT1. PRMT8 methylated the individual histones H2A, H3.3, and H4, with the strongest radiolabeling observed on H2A (Fig. 8 and Fig. S9). Also unlike PRMT1 (Fig. 2), arginine methylation of the Ac-H4(1–20) peptide by PRMT8 was not detectable under this reaction condition. In the presence of the histone octamer, histone H4 alone was strongly methylated by PRMT8, and the level of arginine methylation appeared comparable to the reaction with only H2A as the substrate (Fig. 8*A*, *lane 3*). Consistent with the previous results for all the other PRMTs we examined, PRMT8 did not appear to methylate the mononucleosome.

### The PRMTs do not methylate the HeLa mononucleosomes

**Figure 9:**
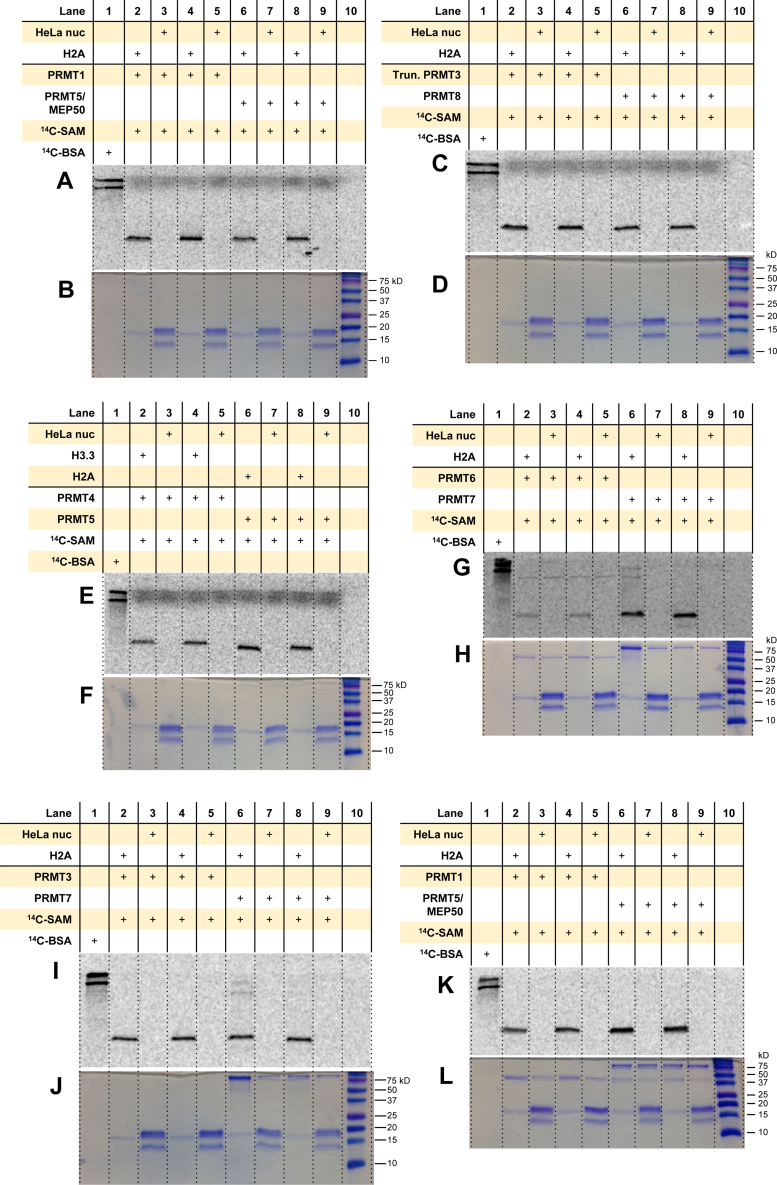
**Comparison of arginine methylation of histones and HeLa mononucleosomes by PRMTs.***A* and *B*, reactions were held for 30 min at 30 °C with 0.05 μM PRMT1 or PRMT5/MEP50, 5 μM [^14^C]SAM, and 1 μM of substrate. *C* and *D*, reactions were held for 30 min at 30 °C with 0.05 μM PRMT8 or truncated PRMT3, 5 μM [^14^C]SAM, and 1 μM of substrate. *E* and *F*, reactions were held for 1 h at 30 °C with 0.05 μM PRMT4 or 0.2 μM PRMT5, 5 μM [^14^C]SAM, and 1 μM of substrate. *G* and *H*, reactions were held for 3 h at 30 °C with 0.5 μM PRMT6 or PRMT7, 5 μM [^14^C]SAM, and 1 μM of substrate. *I* and *J*, reactions were held for 30 min with 0.05 μM PRMT3 or 0.5 μM PRMT7 at 30 °C, 5 μM [^14^C]SAM, and 1 μM of substrate. *K* and *L*, reactions were held for 30 min at 30 °C with 0.5 μM PRMT1 or PRMT5/MEP50, 5 μM [^14^C]SAM, and 1 μM of substrate. *A* and *K*, phosphor images of radiolabeled proteins after 72 h in the dark. *C*, *E*, *G*, and *I*, phosphor images after 96 h in the dark. *B*, *D*, *F*, *G*, *J*, and *L*, Coomassie blue protein staining. [^14^C]BSA and a protein ladder were used in lanes 1 and 10 in all gels as a radiolabeled protein marker and a protein MW ladder, respectively. [^14^C]BSA, [^14^C]-labeled bovine serum albumin; [^14^C]SAM, SAM-(methyl-^14^C); MW, molecular weight; PRMT, protein arginine methyltransferase.

To determine whether the source of mononucleosomes matters in PRMT activity tests, we decided to use the mononucleosomes extracted from HeLa cells. As a control and for comparison, any arginine methylation of the HeLa mononucleosomes by the PRMTs was compared against their preferred individual histone substrate (*i.e.*, H2A for PRMT1, PRMT3, PRMT5, PRMT6, PRMT7, PRMT8 and H3.3 for PRMT4). For all the PRMTs examined, we did not observe any arginine methylation of the HeLa mononucleosomes (Fig. 9). Automethylation of PRMT6 was consistently observed, in agreement with previous reports (Fig. 9*G*) (67, 68, 69, 70). The gel-based assay was repeated with PRMT7 to determine if the PRMT7 automethylation observed in Figure 9*G* was real. Upon repeating the reaction with a new stock (same lot no. 2135) of PRMT7, we only observed arginine methylation of H2A by PRMT7 without any additional radiolabeling of higher molecular weight proteins (Fig. S10).

### High salt does not enable arginine methylation of mononucleosomes by PRMT1 and PRMT5/MEP50

**Figure 10:**
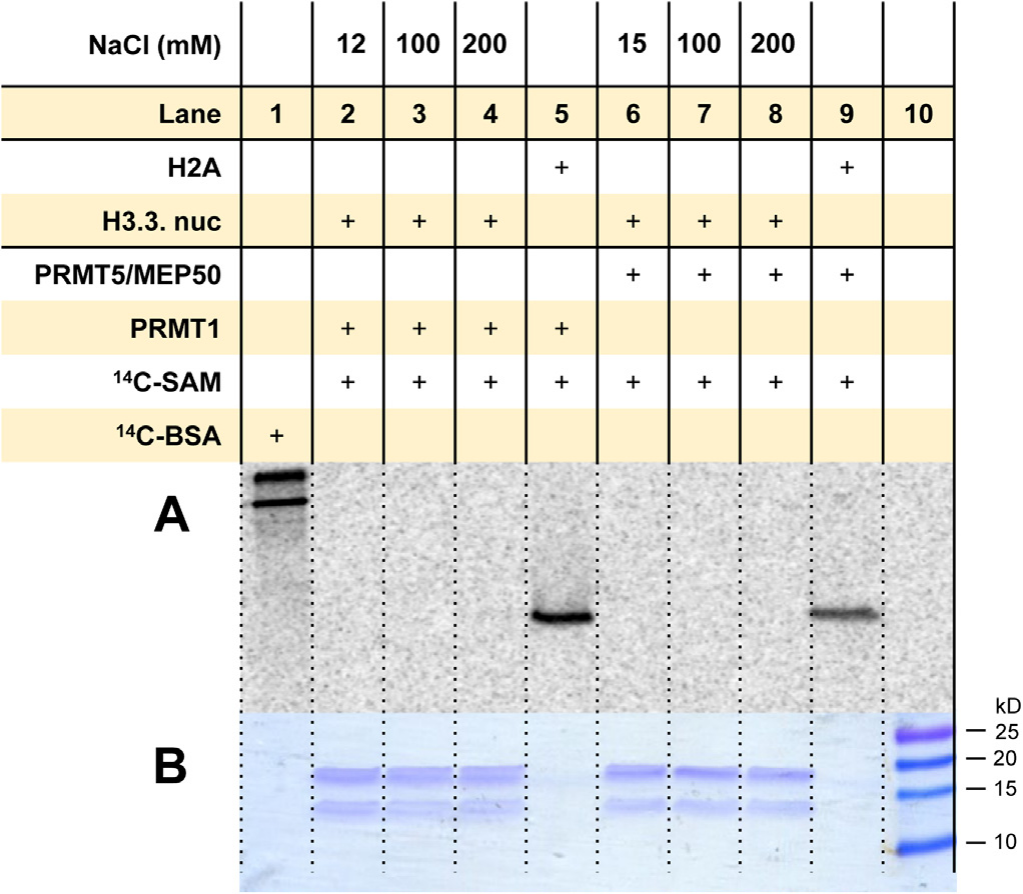
**Arginine methylation by PRMT1 and PRMT5/MEP50 in the presence of low to high salt (NaCl) concentrations.** Reactions were held for 1 h at 30 °C with 0.05 μM PRMT1 or PRMT5/MEP50, 5 μM [^14^C]SAM, and 1 μM of substrate. The final NaCl concentration for reactions with PRMT1 was 12, 100, and 200 mM. The final NaCl concentration for reactions with PRMT5/MEP50 was 15, 100, and 200 mM. Lanes 5 and 9 are the experimental controls to show normal activity of PRMT1 and PRMT/MEP50, measured at 13 and 17 mM of NaCl, respectively. *A*, phosphor image of radiolabeled proteins after 72 h in the dark. *B*, Coomassie blue protein staining. [^14^C]BSA and a protein ladder were used in lanes 1 and 10 as a radiolabeled protein marker and a protein MW reference, respectively. [^14^C]BSA, [^14^C]-labeled bovine serum albumin; [^14^C]SAM, SAM-(methyl-^14^C); MW, molecular weight; PRMT, protein arginine methyltransferase.

After observing the absence of arginine methylation activity of PRMTs on the mononucleosomes, we wondered if this was due to the relatively low salt (NaCl) concentrations in the final reaction mixture (*e.g.*, 12 mM NaCl). The negatively charged phosphate backbone of DNA can form electrostatic interactions with the positively charged histones, especially with the lysine-and arginine-rich N-terminal histone tails (71). Mangenot *et al.* (72) reported an “extended” conformation model, with the histone tails projecting away from the nucleosome core, at high salt concentrations (200 mM NaCl and higher) based on their small angle X-ray scattering data on chicken and calf nucleosome core particles. Therefore, we decided to increase the NaCl concentrations in the reaction to promote the “extended” histone tail conformation to enhance substrate recognition by the PRMTs. As the representative type I and II PRMTs, PRMT1 and PRMT5/MEP50 were chosen in this assay. The reactions were conducted at low salts (based on the NaCl concentrations in the earlier reactions), 100 mM, and 200 mM NaCl concentrations. However, we still did not observe any arginine methylation of the mononucleosomes, at all the salt concentrations: low salt (12 mM NaCl with PRMT1 and 15 mM NaCl with PRMT5), 100 mM, or 200 mM NaCl (Fig. 10 and Fig. S11). Histone H2A was used as a positive control, and its methylation was clearly observed (Fig. 10, *lanes 5* and *9*), although the salt concentration was low (13.4 mM NaCl with PRMT1 and 16.5 mM NaCl with PRMT5).

To exclude the pitfall that the high salt concentrations (100 mM and 200 mM NaCl) were detrimental to PRMT1 or PRMT5/MEP50 enzyme activity, we performed a control experiment with simply histone H4 protein at low (10 mM) and high (100 and 200 mM NaCl) concentrations (Fig. S12). For both PRMT1 and PRMT5/MEP50, arginine methylation levels of H4 were not affected by high concentrations of NaCl. This result clarified that high concentrations of NaCl would not affect enzyme activity of PRMT1 or PRMT5/MEP50. Therefore, the inability to methylate mononucleosomes is an intrinsic property of the tested PRMTs.

### Tetra-acetylation of the histone H4 tail does not enable arginine methylation of mononucleosomes by PRMT1 and PRMT5/MEP50

**Figure 11:**
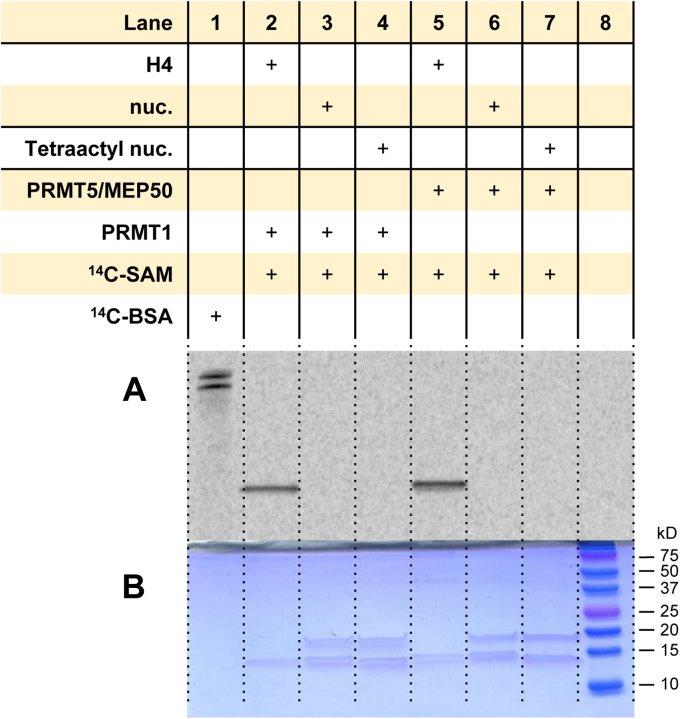
**Arginine methylation by PRMT1 and PRMT5/MEP50 in tetra-acetylated H4 mononucleosome.** Reactions were held for 30 min at 30 °C with 0.05 μM PRMT1 or PRMT5/MEP50, 5 μM [^14^C]SAM, and 1 μM of substrate. *A*, phosphor image of radiolabeled proteins after 72 h in the dark. *B*, Coomassie blue protein staining. [^14^C]BSA and a protein ladder were used in lanes 1 and 8 as a radiolabeled protein marker and a protein MW reference, respectively. [^14^C]BSA, [^14^C]-labeled bovine serum albumin; [^14^C]SAM, SAM-(methyl-^14^C); MW, molecular weight; PRMT, protein arginine methyltransferase.

Although arginine methylation of mononucleosomes by the PRMTs was not detected under all the experimental concentrations, it could be possible that hyperacetylation of the N-terminal tail of histone H4 (*i.e.*, H4K5ac, H4K8ac, H4K12ac, and H4K16ac) may enable arginine methylation of mononucleosomes by the PRMTs. Especially, it has been reported that acetylation of the N-terminal H4 tail affects PRMT1 and PRMT5/MEP50-mediated H4R3 methylation (49, 52). To examine whether hyperacetylation of H4 can promote PRMT-catalyzed arginine methylation of mononucleosomes, we conducted a methylation assay using a recombinant mononucleosome containing tetra-acetylated histone H4 as the substrate. Recombinant histone H4 protein was used as a positive control (Fig. 11, *lanes 2* and *5*), and the unmodified mononucleosome was used as a negative control (Fig. 11, *lanes 3* and *6*). Based on the phosphorimaging results, there was no methylation of mononucleosomes observed, even when the histone H4 tail was present in the hyperacetylated form. We summarized the results of all the methylation experiments in Table 1.

**Table 1:** Summary of arginine methylation of histones, octamer, and mononucleosomes by PRMTs

PRMT enzyme	H2A	H2B	H3.3	H4	Octamer	Recombinant mononucleosome	HeLa mononucleosome
PRMT1	++	—	—	+	H4 > H2A	—	—
Truncated PRMT3	++	—	—	+	—	—	—
PRMT3	++	—	—	+	H4	—	—
CARM1	++	++	+++	+	H3.3	—	—
PRMT5	++	—	—	+	H4	—	—
PRMT5/MEP50	++	—	—	+	H4	—	—
PRMT6	+	—	+	+	H4 > H2A, H3.3	—	—
PRMT7	++	+	—	+	H4	—	—
PRMT8	+++	—	+	++	H4	—	—

Plus signs (+ low, +++ high) indicate relative substrate preference among the individual histones.

(—) indicates that arginine methylation was not observed.

### Nucleosome inhibits methylation of histone H4 by PRMT1 and PRMT5/MEP50

**Figure 12:**
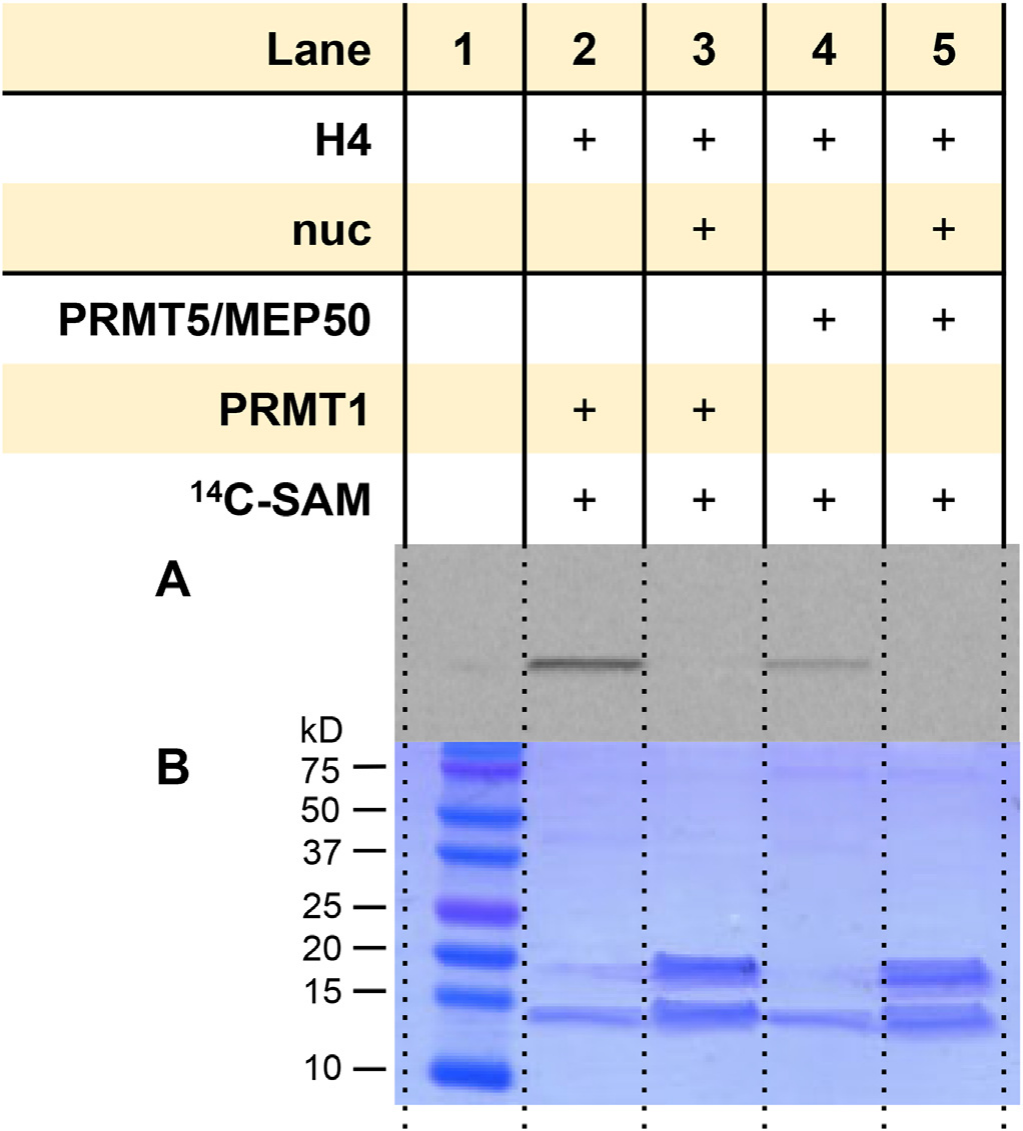
**Arginine methylation of H4 protein by PRMT1 and PRMT5/MEP50 in the presence and absence of recombinant mononucleosomes.** Reactions were held for 30 min at 30 °C with 0.05 μM PRMT1 or PRMT5/MEP50, 5 μM [^14^C]SAM, and 1 μM of nucleosome or reaction buffer. *A*, phosphor image of radiolabeled proteins after 72 h in the dark. *B*, Coomassie blue protein staining. Lane 1 was used as a protein MW reference. [^14^C]BSA, [^14^C]-labeled bovine serum albumin; [^14^C]SAM, SAM-(methyl-^14^C); MW, molecular weight; PRMT, protein arginine methyltransferase.

Because there was no detectable arginine methylation of nucleosomes by PRMT1 or PRMT5, we wondered if the presence of nucleosome would affect the methylation of free histones by PRMT1 and PRMT5/MEP50. To examine the influence of nucleosomes, we tested the methylation level of recombinant histone H4 in the presence (Fig. 12, *lanes 2* and *4*) or the absence of recombinant mononucleosomes (Fig. 12, *lanes 3* and *5*). The result clearly showed that methylation of histone H4 by PRMT1 and PRMT5/MEP50 was abolished in the presence of the nucleosome.

To understand the mechanism for the inhibitory effect of nucleosome on histone H4 methylation by PRMT1, we performed protein interaction assays using the native PAGE (*i.e.*, regular SDS-PAGE without the addition of SDS). Under this condition, protein structures and protein–protein interactions are retained. On the native PAGE gel (Fig. S13), the PRMT1 protein and nucleosome were both clearly visible (*lanes 1* and *3*), likely owing to their negative charges in a running buffer of pH 8 (PRMT1 has a pI of 5.81, and the nucleosome is DNA bound). On the other hand, the recombinant H4 protein (*lane 2*) could not migrate onto the native gel because histone H4 was highly positively charged (pI 11.4). *Lane 4* showed that once PRMT1 was mixed with H4, the PRMT1 protein band disappeared on this gel, ascertaining formation of PRMT1–H4 interactive complex. But, the migrations of PRMT1 and the nucleosome were not mutually affected, highlighting there was no significant binding between PRMT1 and the mononucleosome. Therefore, we concluded that the nucleosome inhibited PRMT1 activity by not binding the enzyme, but there might be some other possibilities.

## Discussion

Arginine methylation of nucleosomal histones is an important regulatory mechanism for gene transcription (6), and these modifications have been observed on the histone tail region, globular domain, and C-terminal region. Many of the PRMTs (*e.g.*, PRMT1, PRMT3, PRMT4, PRMT5, PRMT6, and PRMT7) have been reported to catalyze the formation of MMA, ADMA, and/or SDMA marks on the core histones (15, 30, 35, 42, 45). While previous studies have derived valuable information about how PRMTs are regulated by the substrate sequence and local PTMs on histone-based peptide substrates (17, 47, 49, 50, 51, 52, 73, 74, 75), peptide substrates lack the *in vivo* structural contexts of the histone octamer and the basic unit of chromatin, the nucleosome. To examine the impact of the substrate's macromolecular contexts on arginine methylation, we performed radioactive biochemical assays with recombinant PRMTs and recombinant human full-length histones, histone octamers, and mononucleosomes. Consistent with other observations (42, 51), we observed that all the tested PRMTs demonstrate substrate specificity differences that are dependent on the context of the histones (Table 1). All the PRMTs methylate at least two of the full-length histones individually. Yet, most of the PRMTs (PRMT1, PRMT3, PRMT5, PRMT6, PRMT7, and PRMT8) prefer to methylate H4 in the context of the histone octamer. The only exception is PRMT4/CARM1, which prefers to methylate H3 in the histone octamer even though PRMT4 is able to methylate all the individual full-length recombinant histones. In our hands, we did not observe arginine methylation of mononucleosomes by any of the PRMTs examined, which coincides with the previous reports (24, 43).

Consistent with the earlier reports, PRMT1 and PRMT4 can methylate more than one histone individually, yet these enzymes prefer to methylate mainly histone H4 or histone H3, respectively, in the histone octamer (25). Even though Chen *et al.* (25) used mouse recombinant PRMT4/CARM1, our results are similar to theirs in that human recombinant PRMT4 methylated the individual histones H3.3 > H2A, H2B > H4, and that PRMT4 preferred to methylate H3 in the histone octamer. In consistence, PRMT1 demonstrates a preference for methylating histone H4 in the histone octamer (25, 42). What remains puzzling with PRMT1 is the report that the enzyme is capable of methylating oligonucleosomes from 6C2 cells (16), and yet we did not observe any arginine methylation of the recombinant or extracted mononucleosomes by all the tested PRMTs. It is unclear if those oligonucleosomes used in the previous study possess certain structural characteristics that can promote PRMT1-catalyzed arginine methylation. Another puzzling aspect is that Leal *et al.* (76) recently detected H3 methylation by PRMT1 with an *in vitro* biochemical assay. This is quite surprising because previous studies did not find any H3 methylation activity by PRMT1 (17, 18, 25). Upon a close examination of our phosphorimaging data, it appears that there is a very faint band in the H3.3 reaction sample with PRMT1 (Fig. S1, *lane 5*). Nonetheless, even if PRMT1 indeed methylates histone H3, the labeling is significantly weaker in comparison to H4.

We initially used truncated PRMT3 (residues 211–531, PDB entry: 2FYT) in this study because this was readily available, and our previous study examined how PTMs on histone H4 could regulate PRMT3 catalysis (49). The N-terminal region of PRMT3 may serve an important role for substrate engagement of the histone substrates. It has been previously reported for rat recombinant PRMT3 that the N-terminal acidic amino acid–rich domain (1–194) containing the zinc finger motif (48, 49, 50, 51, 52, 53, 54, 55, 56, 57, 58, 59, 60, 61, 62, 63, 64, 65, 66, 67, 68, 69) is important for substrate specificity (60), and our truncated PRMT3 is missing the zinc finger domain (48–71 human). We determined whether the N-terminal region of PRMT3 is important for substrate recognition of different histone substrates. Overall, truncated and full-length PRMT3 demonstrated the same preferences for the individual histones H2A and H4. However, the level of histone octamer methylation was much higher in the presence of the full-length PRMT3 than the truncated PRMT3 (Fig. 3). This validates that the N-terminal region is indeed important for substrate engagement. Also, our results are consistent with another study that demonstrates FLAG-hemagglutinin-tagged PRMT3 heavily labels histone H4 within the octamer (42). While PRMT3 is often found localized to the cytoplasm (60, 67), there may be cases that result in PRMT3 translocation to the nucleus as observed with the treatment of human embryonic kidney 293 cells with palmitic acid (77). Hence, there may be other physiological contexts for PRMT3 to methylate nuclear histones that have yet to be discovered.

Both PRMT5 and PRMT5/MEP50 methylated H2A and H4 in the pure protein form. A particular notion is that the methylation activity of PRMT5/MEP50 (but not PRMT5 alone) was much higher on H2A than H4 (Figs. S5 and S6), coinciding well with previous results (51, 65). This would support that MEP50 strongly affects the substrate specificity of PRMT5. Interestingly, even though PRMT5 is type II, it shares the same substrate preference for H4 in the histone octamer as type I enzymes PRMT1, PRMT3, PRMT6, PRMT7, and PRMT8. Also, the fact that PRMT5 does not appear to methylate the mononucleosome is consistent with previous observations (51, 65). PRMT5 was previously shown unable to methylate recombinant mononucleosomes unless DNase I is present (65). Thus, substrate contexts strongly affect arginine methylation activity of PRMT5. Yet, one report showed that PRMT5 can methylate histone nucleosomes extracted from K562 cells (30). Also, some studies reported that PRMT5 methylates histone H3 (32, 33, 40, 65, 78). In our study, we observed only a very weak band of radiolabeled H3.3 by PRMT5/MEP50, and the absence of MEP50 abolished this faint methylation (Fig. 5*A* and Fig. S5). These differences may be explained by differences in exposure time, reaction conditions, and/or the presence of PTMs on the histones that might stimulate PRMT5 activity (*e.g.*, H4K5ac promotes H4R3 methylation by PRMT5) (49, 51, 52).

We observed that PRMT6 shows weak automethylation, and this is consistent with previous reports (67, 68). Slightly different than other reports, we observed that human recombinant PRMT6 methylated three histones: H2A, H3.3, and H4, with relatively close intensities. Yet, the arginine methylation by PRMT6 was dominantly on histone H4 in the histone octamer, which was also observed by Casadio *et al.* (42). Others have found that human recombinant PRMT6 methylated H3 and H2A > H4 > H2B, and PRMT6 demonstrates substrate preference for H4 and H3 in the bulk histones (36). Methylation of H3 is expected as it was previously shown that PRMT6 is the major enzyme for H3R2me2a formation (35), and PRMT6 also methylates H3R42 (42). The slight differences in results may be owing to the source of the histones, since others used histones extracted from calf thymus while Casadio *et al.* (36) and we used recombinant human histones. Consistently though, we and others did not observe arginine methylation of nucleosomes by PRMT6.

Based on the study of amino acid analysis of the methylated histones by PRMT7 by Feng *et al.* (45), they observed that PRMT7 methylates H2B > H3.3, H2A > H4 in a 20-h reaction. In this study, we observed that PRMT7 strongly methylated H2A, H2B, and H4. Both H4 and H2B contain the RXR motif (R17 and R19 in H4; R29, R31, and R33 in H2B) that is favored by PRMT7, which resonates that they are good substrates of PRMT7. This is particularly demonstrated in a recent study showing that the H2B (23, 24, 25, 26, 27, 28, 29, 30, 31, 32, 33, 34, 35, 36, 37) peptide was the strongest substrate of PRMT7 compared with a panel of H3 and H4 peptides (79). Lack of H2B methylation in the histone octamer indicates that the RXR motif of H2B was inaccessible to PRMT7 in the octamer context. Although histone H3 methylation by PRMT7 was reported previously (45), we only observed a very faint band of H3.3 methylation, barely above the background level (Fig. 7). The difference in results may be attributed to the fact that we used recombinant human PRMT7, whereas the previous study used mouse recombinant PRMT7. The sequence identity between human and mouse PRMT7 is about 85% (UniProt alignment of Q9NVM4 and Q922X9). A comparative study between the two recombinant proteins would be needed to confirm this difference in substrate preference among species. Furthermore, Szewczyk *et al.* (79) found that sequence extension on the carboxyl end hinders the methylation of the N-terminal H3 peptide, which may explain why the methylation of the full-length H3 protein by PRMT7 is very weak.

PRMT8 shares the highest sequence identity with PRMT1 (80%) among the PRMT family members (66). Yet unlike PRMT1, we observed that PRMT8 appears to methylate H3.3 in addition to H2A and H4, though at less intensity. While PRMT8 is anchored to the plasma membrane because of its N-terminal myristoylation, a few reports describe cases in which endogenous PRMT8 or PRMT8 isoforms are localized to the nucleus (80, 81). We used recombinant human PRMT8 (variant 1 isoform 1, residues 1–394) with the N-terminal Gly, which is subject to myristoylation *in vivo* (20). It would be interesting to see if the other PRMT8 isoforms exhibit a similar substrate preference with histones as compared with the canonical PRMT8. Moreover, future studies would be warranted to examine how arginine methylation in different histones alters when the PRMT8 isoforms are knocked down or knocked out in mammalian cells.

Acetylation of lysine residues in the H4 N-terminal histone tail is expected to neutralize the positive charges, reduce its interaction with the nucleosomal DNA, and increase its exposure to histone-modifying enzymes. Yet, we did not observe any arginine methylation of the tetra-acetylated nucleosomes by PRMT1 or PRMT5. On one hand, the results are in agreement with what we would expect for PRMT1 given that a peptide with H4K5ac, H4K8ac, H4K12ac, and H4K16ac is a very poor substrate for PRMT1 (52). However, on the other hand, no methylation by PRMT5 of the hyperacetylated mononucleosome is discordant with the previous observation that a H4 peptide with K5ac, K8ac, K12ac, and K16ac is a better substrate of PRMT5 than the H4 peptide (18). Apparently, additional factors play modulatory roles in mediating PRMT5 interaction with the nucleosome substrate. In terms of effects of nucleosome acetylation on histone arginine methylation, Xu *et al.* (82) showed that acetylated nucleosome by p300 is a better substrate of CARM1.

**Figure 13:**
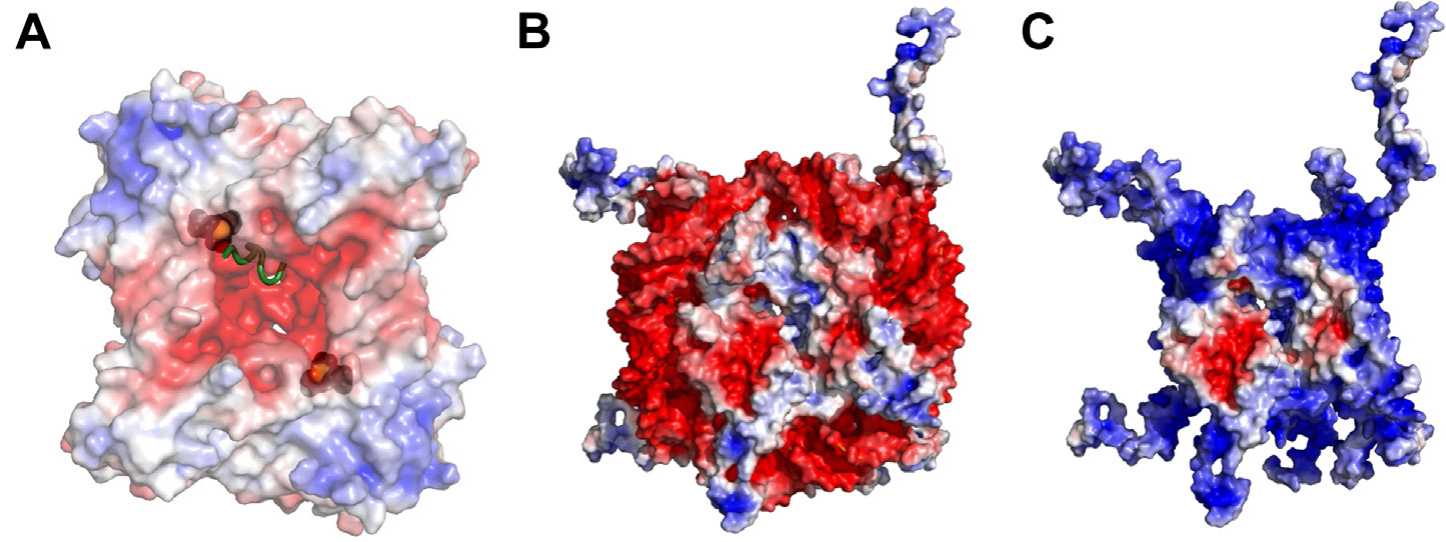
**Charge–charge interaction is important for the substrate recognition by PRMTs.** Electrostatic surface potentials are calculated by the adaptive Poisson–Boltzmann solver and ramped from *red* to *blue* (anionic to cationic). *A*, surface potential of PRMT1 (Protein Data Bank [PBD]: 6NT2) is shown in transparency and the buried SAH molecules shown as *black spheres*. The sulfur atom from SAH is shown as *yellow sphere* to indicate the active site. A substrate peptide sequence from the superimposed CARM1 structure (PDB ID: 5DWQ) is shown as a *green cartoon* to indicate the entry of the arginyl peptide. *B*, surface potential of histone nucleosome (PDB ID: 1KX5). *C*, the surface potential of octamer (PDB: 1KX5 without DNA). CARM1, coactivator-associated arginine methyltransferase 1; PRMT, protein arginine methyltransferase.

The inability of the PRMTs in methylating nucleosomes deserves much mechanistic investigation. We examined the surface potential of PRMT1 and found that the inner ring region of the homodimer where the active site and the entrance of the arginyl peptide reside is highly acidic (*i.e.*, negatively charged) (Fig. 13). With regard to the substrate, the nucleosome possesses a huge negative potential mainly owing to the bound DNA; conversely, the histone octamer core is abundantly cationic. We propose that PRMT–substrate interaction is significantly primed by the attractive electrostatic contacts between the acidic surface of PRMTs and the alkaline surface of their substrates, and under this interactive context, specific bindings come into play to fine tune individual arginine site specificity (Fig. S14). The electrostatic repulsion between the highly negatively charged nucleosome and the negatively charged substrate-binding patch in PRMTs is the most plausible cause for our observation of no methylation activity on the nucleosome. The scenario reverses when the histone octamer comes into contact with PRMTs: the electrostatic attraction primes the proximity between the substrate and the enzyme, which subsequently allows the substrate arginine site to orient toward the PRMT active site. Furthermore, we found that the nucleosome not only is an inert substrate of PRMT1 and PRMT5 but also the presence of nucleosome also inhibits their activity for free H4 methylation. Previously, we have found that the presence of DNA fragments in the reaction buffer inhibits PRMT1 activity for H4 methylation as well (52). It can be envisioned that the negatively charged DNA macromolecule acts as an acidic cloud that shields the arginine substrate out of recognition by PRMTs. Analogously, the H4 peptide can be bound by certain anionic small-molecule ligands, and the binding consequently shields the substrate arginine site from recognition and methylation by PRMT1 (83, 84, 85). Consistent with our study, Ho *et al.* (51) showed that PRMT5 has an acidic patch nearby the active site, which repulses the negatively charged nucleosome to approach. Hence, unwrapping the DNA from the nucleosome to expose certain areas of the histone fold domains could lead to permissive arginine methylation. Indeed, Burgos *et al.* (65) found that PRMT5/MEP50 is able to methylate the nucleosome after the DNA digestion by DNase I. Our electrostatic priming model is further supported by the observation that phosphorylation of residues near the arginine sites in histones introduces negative charges to the substrates and abolishes their methylation by PRMTs (13, 53). Altogether, it appears to be a common theme that the alkaline histone substrates are shielded by negatively charged biomolecules (*e.g.*, DNA and small molecules), which prevents the histones from being recognized and modified by the PRMT enzymes.

Arginine methylation marks in the nucleosomal histones are widely observed. Our results showing that the recombinant or extracted nucleosomes are not a working substrate of PRMTs raise a profound biological question of how histone arginine methylation is introduced inside the cell. One possibility could be that the core histones are arginine methylated prior to their assembly onto chromatin DNAs. In this regard, arginine methylation may play other roles in regulating histone properties such as cytosol–nucleus transport and histone–chaperone interaction. Indeed, PRMT5/MEP50 is found to almost exclusively reside in the cytoplasm of embryonic stem cells where it selectively methylates cytosolic H2A (31). Also, PRMT7 is predominantly cytosolic (79), and PRMT8 is associated with the plasma membrane (20), both of which are capable of histone methylation. A particular notion is that, despite belonging to the same class I methyltransferase family with PRMTs, the histone H3 methyltransferase DOT1L methylates nucleosomes as a substrate but not isolated histone H3 protein or peptide (86, 87). The DOT1L catalytic domain contains multiple anchors that dock DOT1L on the surface of nucleosomes, including a lysine-rich region that binds nucleosomal DNA, and DNA binding stimulates its H3K79 methylation activity (88, 89). In contrast, no PRMTs appear to possess any DNA-binding domains. This raises another possibility that additional protein partners are required for PRMTs to access and methylate the nucleosome. Especially, transcriptional factors may bind and recruit PRMTs to specific gene loci, thereby enabling their activity of nucleosome methylation. Under this scenario, it is conceivable that such “recruiter” proteins may transiently unwrap the DNA of the nucleosome to expose the N-terminal tails or even the globular domain of the core histones for arginine methylation by PRMTs. Extracting PRMT complexes from their nascent environment would be needed to test this hypothesis. One study shows that the transcriptional repressor Blimp1 recruits PRMT5 to methylate arginine 3 on histone H4 and/or H2A tails at the *Dhx38* locus (29); however, it is unknown if the Blimp1–PRMT5 complex is capable of methylating nucleosomes. Moreover, it is not clear what kinds of PTMs on the histones in the nucleosome are capable of promoting arginine methylation of nucleosomes by the PRMT family members. Although we did not find any positive effect of H4 tetra-acetylation on PRMT1 and PRMT5 activity in nucleosomes, a previous study showed that nucleosome acetylation by the promiscuous lysine acetyltransferase p300 greatly activates nucleosome methylation by CARM1 (82). Albeit costly, detailed effects of PTMs could be elucidated by screening a large panel of nucleosomes containing various PTMs against individual PRMT members. Overall, dissecting the dynamic mechanisms that enable PRMT-catalyzed arginine methylation of the nucleosomal histones is of essential importance for understanding PRMT function in epigenetic regulation.

In conclusion, we sought to determine how the substrate context regulates histone arginine methylation by the PRMT family members. Our work demonstrated that PRMT1, PRMT3, PRMT4, PRMT5, PRMT6, PRMT7, and PRMT8 are capable of methylating at least two histones individually, yet most of the PRMTs display a preference for one histone substrate in the context of the human recombinant histone octamer: PRMT1, PRMT3, PRMT5, PRMT6, PRMT7, and PRMT8 preferred to methylate histone H4 in the histone octamer, and only PRMT4 preferred to methylate H3 in the histone octamer. All the PRMTs examined in this study did not methylate human mononucleosomes, either the reconstituted or the one extracted from HeLa cell nuclear lysates. Even with tetra-acetylated mononucleosomes, we did not observe any arginine methylation by PRMTs. Further investigation is warranted to better understand structural mechanisms that dictate the substrate preference in PRMT biochemistry. Overall, this work corroborates the divergence in substrate preference among individual PRMT members, and highlights the importance of the substrate context for impacting arginine methylation in PRMT biology.

## Experimental procedures

### Chemical reagents

PMSF, kanamycin, ampicillin, and IPTG were purchased from Gold Biotechnology. The *N-α*-Fmoc–protected amino acids were purchased from Novabiochem, Chem-Impex International, Inc, or ChemPep, Inc. SAM-(methyl-^14^C), abbreviated as [^14^C]SAM, was purchased from PerkinElmer. All other chemical reagents were purchased from Fisher Scientific, Oakwood Chemicals, Acros Organics, Sigma–Aldrich, Alfa Aesar, BDH, Research Products International Corp, Macron Fine Chemicals, Bio-Rad, EpiCypher, Inc, or J. T. Baker.

### Protein expression and purification

Human recombinant His-tag PRMT1 (residues 11–353, pET28b(+) vector), His-tag truncated PRMT3 (residues 211–531, pET28a-LIC vector), His-tag PRMT3 (residues 1–544, pReceiver-B01 vector), His-tag PRMT6 (residues 1–375, pET28a vector), and His-tag PRMT8 (residues 1–394, pET100 vector) were expressed from *Escherichia coli* and purified by immobilized metal (nickel) chelation chromatography as previously described (49). Human recombinant His-tag PRMT5 was coexpressed with His-tag MEP50 using the Bac-to-Bac baculovirus expression system (Invitrogen, Life Technologies) as previously described (53). Human recombinant FLAG-tag (N-term) PRMT4/CARM1 was purchased from Millipore (catalog no.: 14-1048, lot no. 3164605, 585 amino acids, 63.46 kDa). Human recombinant His-tag PRMT7 (residues 2–692) was purchased from Reaction Biology (catalog no. HMT-21-382, lot no. 2135). Full-length human recombinant PRMT5 alone (without MEP50) was purchased from Reaction Biology (catalog no. HMT-21-172, lot no. 1451). Human recombinant full-length histones H2A (catalog no. M2502S), H2B (catalog no. M2505S), H3.3 (catalog no. M2507S), and H4 (catalog no. M2504S) were purchased from New England Biolabs. The following items were purchased from EpiCypher, including human recombinant histone H3.3 octamer (catalog no. 16-8012, lot no. 18253001), H3.3 mononucleosomes (catalog no. 16-0012, lot no. 17218003), HeLa nucleosomes (catalog no. 16-0002, lot no. 18082012), and the H4 tetra-acetyl nucleosome (catalog no. 16-0313, lot no. 18124001) and its control mononucleosomes (catalog no. 16-0009, lot no. 19344014-01).

### Peptide synthesis

Peptides Ac-H4(1–20) (sequence Ac-SGRGKGGKGLGKGGAKRHRK-OH), Ac-H2A(1–21) (sequence Ac-SGRGKQGGKARAKAKTRSSRA-OH), and Ac-H3(1–20) (sequence Ac-ARTKQTARKSTGGKAPRKQL-OH) were synthesized using Fmoc solid-phase peptide synthesis and purified as previously described (49). Peptide concentrations for Ac-H2A(1–21) and Ac-H3(1–20) were determined by weight. The concentration of Ac-H4(1–20) was determined with ^1^H NMR using 2,2-dimethyl-2-silapentane-5-sulfonate sodium salt as an internal standard as previously described (49). See Supporting information (Fig. S15) for ^1^H NMR spectra and concentration adjustment factor.

### Radioactive methylation (gel-based) assay

Peptides, histones, nucleosomes, and PRMTs were kept on ice and diluted in reaction buffer (50 mM Hepes, pH 8, 10 mM NaCl, 0.5 mM EDTA, and 0.5 mM DTT) to prepare working solutions. About 6 μl of [^14^C]SAM was combined with 6 μl of peptide, 6 μl of an equimolar amount of histone, or 6 μl of reaction buffer before initiating the reaction with 18 μl of PRMT. The final concentrations of each component in the reactions were 1 μM of peptide/histone, 5 μM of [^14^C]SAM, and 0.05, 0.5, or 0.2 μM of PRMT. Reactions proceeded for 30 min at 30 °C, unless noted otherwise, and quenched with 6 μl of 6× Tris–tricine gel loading buffer (200 mM Tris–HCl, pH 6.8, 40% glycerol, 14% SDS, 300 mM DTT, and 0.06% Coomassie blue) and pulse vortexed. All samples are heated for 10 min at 95 °C. About 30 μl of reaction sample was loaded into each well of a 16% SDS-PAGE gel. To vary the NaCl concentrations, a separate reaction buffer was prepared at different NaCl concentrations (851 mM–1.87 M NaCl) and diluted with the mononucleosomes or histones prior to adding radiolabeled SAM and initiating the reaction with PRMTs. We took into consideration the NaCl from the stock solutions of histones and mononucleosomes, PRMT storage buffers, and reaction buffer. As a molecular weight reference, 7 μl of the Precision Plus Protein Dual Color Standard (Bio-Rad; catalog no. 161-0374) was added to lane 10. Proteins were resolved with 100 V for 10 min followed by 140 V for 75 min in a Tris–tricine running buffer (100 mM Tris, 100 mM tricine, and 0.1% SDS). Gels were soaked in Coomassie staining solution (10% acetic acid, 45% methanol, 3% glycerol, 41.5% water, and 0.5% Coomassie blue) for 20 min and then soaked in destaining solution (10% acetic acid, 45% methanol, 3% glycerol, and 42% water) for 40 min. Gels were dried in a Bio-Rad Gel Dryer (model 583) under vacuum for 2 h on gradient mode at 70 °C. An Amersham Biosciences phosphor screen was exposed to the dried gels in the dark for 72 h before scanning the screens with a GE Storm 865 Phosphor Imager. Gels that did not stain well during the initial 1 h process of staining and destaining were rehydrated after the phosphorimaging to restain the proteins.

## Data availability

The authors confirm that the data supporting the findings of this study are available within the article and its supporting information.

## Supporting information

This article contains supporting information.

## Conflict of interest

The authors declare that they have no conflicts of interest with the contents of this article.

## References

[1] Olins D.E., Olins A.L. (2003). Chromatin history: Our view from the bridge. Nat. Rev. Mol. Cell Biol..

[2] McGhee J.D., Felsenfeld G. (1980). Nucleosome structure. Annu. Rev. Biochem..

[3] Thomas J.O., Kornberg R.D. (1975). An octamer of histones in chromatin and free in solution. Proc. Natl. Acad. Sci. U. S. A..

[4] Luger K., Mader A.W., Richmond R.K., Sargent D.F., Richmond T.J. (1997). Crystal structure of the nucleosome core particle at 2.8 angstrom resolution. Nature.

[5] Strahl B.D., Allis C.D. (2000). The language of covalent histone modifications. Nature.

[6] Molina-Serrano D., Schiza V., Kirmizis A. (2013). Cross-talk among epigenetic modifications: Lessons from histone arginine methylation. Biochem. Soc. Trans..

[7] Huang H., Sabari B.R., Garcia B.A., Allis C.D., Zhao Y. (2014). SnapShot: Histone modifications. Cell.

[8] Di Lorenzo A., Bedford M.T. (2011). Histone arginine methylation. FEBS Lett..

[9] Bedford M.T., Clarke S.G. (2009). Protein arginine methylation in mammals: Who, what, and why. Mol. Cell.

[10] Litt M., Qiu Y., Huang S. (2009). Histone arginine methylations: Their roles in chromatin dynamics and transcriptional regulation. Biosci. Rep..

[11] Wysocka J., Allis C.D., Coonrod S. (2006). Histone arginine methylation and its dynamic regulation. Front. Biosci..

[12] Gayatri S., Bedford M.T. (2014). Readers of histone methylarginine marks. Biochim. Biophys. Acta.

[13] Fulton M.D., Brown T., Zheng Y.G. (2018). Mechanisms and inhibitors of histone arginine methylation. Chem. Rec..

[14] Wolf S.S. (2009). The protein arginine methyltransferase family: An update about function, new perspectives and the physiological role in humans. Cell. Mol. Life Sci..

[15] Strahl B.D., Briggs S.D., Brame C.J., Caldwell J.A., Koh S.S., Ma H., Cook R.G., Shabanowitz J., Hunt D.F., Stallcup M.R., Allis C.D. (2001). Methylation of histone H4 at arginine 3 occurs *in vivo* and is mediated by the nuclear receptor coactivator PRMT1. Curr. Biol..

[16] Huang S., Litt M., Felsenfeld G. (2005). Methylation of histone H4 by arginine methyltransferase PRMT1 is essential *in vivo* for many subsequent histone modifications. Genes Dev..

[17] Wang H., Huang Z.Q., Xia L., Feng Q., Erdjument-Bromage H., Strahl B.D., Briggs S.D., Allis C.D., Wong J., Tempst P., Zhang Y. (2001). Methylation of histone H4 at arginine 3 facilitating transcriptional activation by nuclear hormone receptor. Science.

[18] Lee J., Cheng D., Bedford M.T. (2004). Techniques in protein methylation. Methods Mol. Biol..

[19] Yang Y., Lu Y., Espejo A., Wu J., Xu W., Liang S., Bedford M.T. (2010). TDRD3 is an effector molecule for arginine-methylated histone marks. Mol. Cell.

[20] Lee J., Sayegh J., Daniel J., Clarke S., Bedford M.T. (2005). PRMT8, a new membrane-bound tissue-specific member of the protein arginine methyltransferase family. J. Biol. Chem..

[21] Kaniskan H.U., Szewczyk M.M., Yu Z., Eram M.S., Yang X., Schmidt K., Luo X., Dai M., He F., Zang I., Lin Y., Kennedy S., Li F., Dobrovetsky E., Dong A. (2015). A potent, selective and cell-active allosteric inhibitor of protein arginine methyltransferase 3 (PRMT3). Angew. Chem. Int. Ed. Engl..

[22] Allali-Hassani A., Wasney G.A., Siarheyeva A., Hajian T., Arrowsmith C.H., Vedadi M. (2012). Fluorescence-based methods for screening writers and readers of histone methyl marks. J. Biomol. Screen..

[23] Siarheyeva A., Senisterra G., Allali-Hassani A., Dong A., Dobrovetsky E., Wasney G.A., Chau I., Marcellus R., Hajian T., Liu F., Korboukh I., Smil D., Bolshan Y., Min J., Wu H. (2012). An allosteric inhibitor of protein arginine methyltransferase 3. Structure.

[24] Schurter B.T., Koh S.S., Chen D., Bunick G.J., Harp J.M., Hanson B.L., Henschen-Edman A., Mackay D.R., Stallcup M.R., Aswad D.W. (2001). Methylation of histone H3 by coactivator-associated arginine methyltransferase 1. Biochemistry.

[25] Chen D., Ma H., Hong H., Koh S.S., Huang S.M., Schurter B.T., Aswad D.W., Stallcup M.R. (1999). Regulation of transcription by a protein methyltransferase. Science.

[26] Yang G., Zhou C., Wang R., Huang S., Wei Y., Yang X., Liu Y., Li J., Lu Z., Ying W., Li X., Jing N., Huang X., Yang H., Qiao Y. (2019). Base-editing-mediated R17H substitution in histone H3 reveals methylation-dependent regulation of Yap signaling and early mouse embryo development. Cell Rep..

[27] Hatanaka Y., Tsusaka T., Shimizu N., Morita K., Suzuki T., Machida S., Satoh M., Honda A., Hirose M., Kamimura S., Ogonuki N., Nakamura T., Inoue K., Hosoi Y., Dohmae N. (2017). Histone H3 methylated at arginine 17 is essential for reprogramming the paternal genome in zygotes. Cell Rep..

[28] Pollack B.P., Kotenko S.V., He W., Izotova L.S., Barnoski B.L., Pestka S. (1999). The human homologue of the yeast proteins Skb1 and Hsl7p interacts with Jak kinases and contains protein methyltransferase activity. J. Biol. Chem..

[29] Ancelin K., Lange U.C., Hajkova P., Schneider R., Bannister A.J., Kouzarides T., Surani M.A. (2006). Blimp1 associates with Prmt5 and directs histone arginine methylation in mouse germ cells. Nat. Cell Biol..

[30] Zhao Q., Rank G., Tan Y.T., Li H., Moritz R.L., Simpson R.J., Cerruti L., Curtis D.J., Patel D.J., Allis C.D., Cunningham J.M., Jane S.M. (2009). PRMT5-mediated methylation of histone H4R3 recruits DNMT3A, coupling histone and DNA methylation in gene silencing. Nat. Struct. Mol. Biol..

[31] Tee W.W., Pardo M., Theunissen T.W., Yu L., Choudhary J.S., Hajkova P., Surani M.A. (2010). Prmt5 is essential for early mouse development and acts in the cytoplasm to maintain ES cell pluripotency. Genes Dev..

[32] Pal S., Vishwanath S.N., Erdjument-Bromage H., Tempst P., Sif S. (2004). Human SWI/SNF-associated PRMT5 methylates histone H3 arginine 8 and negatively regulates expression of ST7 and NM23 tumor suppressor genes. Mol. Cell. Biol..

[33] Wang L., Pal S., Sif S. (2008). Protein arginine methyltransferase 5 suppresses the transcription of the RB family of tumor suppressors in leukemia and lymphoma cells. Mol. Cell. Biol..

[34] Dong F., Li Q., Yang C., Huo D., Wang X., Ai C., Kong Y., Sun X., Wang W., Zhou Y., Liu X., Li W., Gao W., Liu W., Kang C. (2018). PRMT2 links histone H3R8 asymmetric dimethylation to oncogenic activation and tumorigenesis of glioblastoma. Nat. Commun..

[35] Guccione E., Bassi C., Casadio F., Martinato F., Cesaroni M., Schuchlautz H., Lüscher B., Amati B. (2007). Methylation of histone H3R2 by PRMT6 and H3K4 by an MLL complex are mutually exclusive. Nature.

[36] Hyllus D., Stein C., Schnabel K., Schiltz E., Imhof A., Dou Y., Hsieh J., Bauer U.M. (2007). PRMT6-mediated methylation of R2 in histone H3 antagonizes H3 K4 trimethylation. Genes Dev..

[37] Iberg A.N., Espejo A., Cheng D., Kim D., Michaud-Levesque J., Richard S., Bedford M.T. (2008). Arginine methylation of the histone H3 tail impedes effector binding. J. Biol. Chem..

[38] Kleinschmidt M.A., de Graaf P., van Teeffelen H.A., Timmers H.T. (2012). Cell cycle regulation by the PRMT6 arginine methyltransferase through repression of cyclin-dependent kinase inhibitors. PLoS One.

[39] Stein C., Riedl S., Ruthnick D., Notzold R.R., Bauer U.M. (2012). The arginine methyltransferase PRMT6 regulates cell proliferation and senescence through transcriptional repression of tumor suppressor genes. Nucleic Acids Res..

[40] Migliori V., Muller J., Phalke S., Low D., Bezzi M., Mok W.C., Sahu S.K., Gunaratne J., Capasso P., Bassi C., Cecatiello V., De Marco A., Blackstock W., Kuznetsov V., Amati B. (2012). Symmetric dimethylation of H3R2 is a newly identified histone mark that supports euchromatin maintenance. Nat. Struct. Mol. Biol..

[41] Lorton B.M., Harijan R.K., Burgos E.S., Bonanno J.B., Almo S.C., Shechter D. (2020). A binary arginine methylation switch on histone H3 arginine 2 regulates its interaction with WDR5. Biochemistry.

[42] Casadio F., Lu X.D., Pollock S.B., LeRoy G., Garcia B.A., Muir T.W., Roeder R.G., Allis C.D. (2013). H3R42me2a is a histone modification with positive transcriptional effects. Proc. Natl. Acad. Sci. U. S. A..

[43] Waldmann T., Izzo A., Kamieniarz K., Richter F., Vogler C., Sarg B., Lindner H., Young N.L., Mittler G., Garcia B.A., Schneider R. (2011). Methylation of H2AR29 is a novel repressive PRMT6 target. Epigenetics Chromatin.

[44] Zurita-Lopez C.I., Sandberg T., Kelly R., Clarke S.G. (2012). Human protein arginine methyltransferase 7 (PRMT7) is a type III enzyme forming omega-NG-monomethylated arginine residues. J. Biol. Chem..

[45] Feng Y., Maity R., Whitelegge J.P., Hadjikyriacou A., Li Z., Zurita-Lopez C., Al-Hadid Q., Clark A.T., Bedford M.T., Masson J.-Y., Clarke S.G. (2013). Mammalian protein arginine methyltransferase 7 (PRMT7) specifically targets RXR sites in lysine- and arginine-rich regions. J. Biol. Chem..

[46] Thandapani P., O'Connor T.R., Bailey T.L., Richard S. (2013). Defining the RGG/RG motif. Mol. Cell.

[47] Jain K., Jin C.Y., Clarke S.G. (2017). Epigenetic control via allosteric regulation of mammalian protein arginine methyltransferases. Proc. Natl. Acad. Sci. U. S. A..

[48] Hadjikyriacou A., Yang Y., Espejo A., Bedford M.T., Clarke S.G. (2015). Unique features of human protein arginine methyltransferase 9 (PRMT9) and its substrate RNA splicing factor SF3B2. J. Biol. Chem..

[49] Fulton M.D., Zhang J., He M., Ho M.C., Zheng Y.G. (2017). Intricate effects of alpha-amino and lysine modifications on arginine methylation of the N-terminal tail of histone H4. Biochemistry.

[50] Osborne T.C., Obianyo O., Zhang X., Cheng X., Thompson P.R. (2007). Protein arginine methyltransferase 1: Positively charged residues in substrate peptides distal to the site of methylation are important for substrate binding and catalysis. Biochemistry.

[51] Ho M.C., Wilczek C., Bonanno J.B., Xing L., Seznec J., Matsui T., Carter L.G., Onikubo T., Kumar P.R., Chan M.K., Brenowitz M., Cheng R.H., Reimer U., Almo S.C., Shechter D. (2013). Structure of the arginine methyltransferase PRMT5-MEP50 reveals a mechanism for substrate specificity. PLoS One.

[52] Feng Y., Wang J., Asher S., Hoang L., Guardiani C., Ivanov I., Zheng Y.G. (2011). Histone H4 acetylation differentially modulates arginine methylation by an in cis mechanism. J. Biochem..

[53] Fulton M.D., Dang T., Brown T., Zheng Y.G. (2020). Effects of substrate modifications on the arginine dimethylation activities of PRMT1 and PRMT5. Epigenetics.

[54] Ganesh L., Yoshimoto T., Moorthy N.C., Akahata W., Boehm M., Nabel E.G., Nabel G.J. (2006). Protein methyltransferase 2 inhibits NF-kappaB function and promotes apoptosis. Mol. Cell. Biol..

[55] Szenker E., Ray-Gallet D., Almouzni G. (2011). The double face of the histone variant H3.3. Cell Res..

[56] Pawlak M.R., Scherer C.A., Chen J., Roshon M.J., Ruley H.E. (2000). Arginine N-methyltransferase 1 is required for early postimplantation mouse development, but cells deficient in the enzyme are viable. Mol. Cell. Biol..

[57] Tang J., Frankel A., Cook R.J., Kim S., Paik W.K., Williams K.R., Clarke S., Herschman H.R. (2000). PRMT1 is the predominant type I protein arginine methyltransferase in mammalian cells. J. Biol. Chem..

[58] An W., Kim J., Roeder R.G. (2004). Ordered cooperative functions of PRMT1, p300, and CARM1 in transcriptional activation by p53. Cell.

[59] Barsyte-Lovejoy D., Li F.L., Oudhoff M.J., Tatlock J.H., Dong A.P., Zeng H., Wu H., Freeman S.A., Schapira M., Senisterra G.A., Kuznetsova E., Marcellus R., Allali-Hassani A., Kennedy S., Lambert J.P. (2014). (R)-PFI-2 is a potent and selective inhibitor of SETD7 methyltransferase activity in cells. Proc. Natl. Acad. Sci. U. S. A..

[60] Tang J., Gary J.D., Clarke S., Herschman H.R. (1998). PRMT 3, a type I protein arginine N-methyltransferase that differs from PRMT1 in its oligomerization, subcellular localization, substrate specificity, and regulation. J. Biol. Chem..

[61] Zhang Z., Nikolai B.C., Gates L.A., Jung S.Y., Siwak E.B., He B., Rice A.P., O'Malley B.W., Feng Q. (2017). Crosstalk between histone modifications indicates that inhibition of arginine methyltransferase CARM1 activity reverses HIV latency. Nucleic Acids Res..

[62] Bauer U.M., Daujat S., Nielsen S.J., Nightingale K., Kouzarides T. (2002). Methylation at arginine 17 of histone H3 is linked to gene activation. EMBO Rep..

[63] Jacques S.L., Aquino K.P., Gureasko J., Boriack-Sjodin P.A., Porter Scott M., Copeland R.A., Riera T.V. (2016). CARM1 preferentially methylates H3R17 over H3R26 through a random kinetic mechanism. Biochemistry.

[64] Wilczek C., Chitta R., Woo E., Shabanowitz J., Chait B.T., Hunt D.F., Shechter D. (2011). Protein arginine methyltransferase Prmt5-Mep50 methylates histones H2A and H4 and the histone chaperone nucleoplasmin in Xenopus laevis eggs. J. Biol. Chem..

[65] Burgos E.S., Wilczek C., Onikubo T., Bonanno J.B., Jansong J., Reimer U., Shechter D. (2015). Histone H2A and H4 N-terminal tails are positioned by the MEP50 WD repeat protein for efficient methylation by the PRMT5 arginine methyltransferase. J. Biol. Chem..

[66] Toma-Fukai S., Kim J.D., Park K.E., Kuwabara N., Shimizu N., Krayukhina E., Uchiyama S., Fukamizu A., Shimizu T. (2016). Novel helical assembly in arginine methyltransferase 8. J. Mol. Biol..

[67] Frankel A., Yadav N., Lee J.H., Branscombe T.L., Clarke S., Bedford M.T. (2002). The novel human protein arginine N-methyltransferase PRMT6 is a nuclear enzyme displaying unique substrate specificity. J. Biol. Chem..

[68] Singhroy D.N., Mesplede T., Sabbah A., Quashie P.K., Falgueyret J.P., Wainberg M.A. (2013). Automethylation of protein arginine methyltransferase 6 (PRMT6) regulates its stability and its anti-HIV-1 activity. Retrovirology.

[69] Lakowski T.M., Frankel A. (2008). A kinetic study of human protein arginine N-methyltransferase 6 reveals a distributive mechanism. J. Biol. Chem..

[70] Bonnefond L., Stojko J., Mailliot J., Troffer-Charlier N., Cura V., Wurtz J.M., Cianferani S., Cavarelli J. (2015). Functional insights from high resolution structures of mouse protein arginine methyltransferase 6. J. Struct. Biol..

[71] Walker I.O. (1984). Differential dissociation of histone tails from core chromatin. Biochemistry.

[72] Mangenot S., Leforestier A., Vachette P., Durand D., Livolant F. (2002). Salt-induced conformation and interaction changes of nucleosome core particles. Biophys. J..

[73] Hu H., Luo C., Zheng Y.G. (2016). Transient kinetics define a complete kinetic model for protein arginine methyltransferase 1. J. Biol. Chem..

[74] Obianyo O., Osborne T.C., Thompson P.R. (2008). Kinetic mechanism of protein arginine methyltransferase 1. Biochemistry.

[75] Obianyo O., Thompson P.R. (2012). Kinetic mechanism of protein arginine methyltransferase 6 (PRMT6). J. Biol. Chem..

[76] Leal J.A., Estrada-Tobar Z.M., Wade F., Mendiola A.J.P., Meza A., Mendoza M., Nerenberg P.S., Zurita-Lopez C.I. (2020). Phosphoserine inhibits neighboring arginine methylation in the RKS motif of histone H3. Arch. Biochem. Biophys..

[77] Kim D.I., Park M.J., Lim S.K., Park J.I., Yoon K.C., Han H.J., Gustafsson J.A., Lim J.H., Park S.H. (2015). PRMT3 regulates hepatic lipogenesis through direct interaction with LXR alpha. Diabetes.

[78] Dacwag C.S., Ohkawa Y., Pal S., Sif S., Imbalzano A.N. (2007). The protein arginine methyltransferase Prmt5 is required for myogenesis because it facilitates ATP-dependent chromatin remodeling. Mol. Cell. Biol..

[79] Szewczyk M.M., Ishikawa Y., Organ S., Sakai N., Li F., Halabelian L., Ackloo S., Couzens A.L., Eram M., Dilworth D., Fukushi H., Harding R., Dela Sena C.C., Sugo T., Hayashi K. (2020). Pharmacological inhibition of PRMT7 links arginine monomethylation to the cellular stress response. Nat. Commun..

[80] Kousaka A., Mori Y., Koyama Y., Taneda T., Miyata S., Tohyama M. (2009). The distribution and characterization of endogenous protein arginine N-methyltransferase 8 in mouse CNS. Neuroscience.

[81] Hernandez S., Dominko T. (2016). Novel protein arginine methyltransferase 8 isoform is essential for cell proliferation. J. Cell. Biochem..

[82] Xu W., Chen H., Du K., Asahara H., Tini M., Emerson B.M., Montminy M., Evans R.M. (2001). A transcriptional switch mediated by cofactor methylation. Science.

[83] Feng Y., Xie N., Wu J., Yang C., Zheng Y.G. (2009). Inhibitory study of protein arginine methyltransferase 1 using a fluorescent approach. Biochem. Biophys. Res. Commun..

[84] Feng Y., Li M., Wang B., Zheng Y.G. (2010). Discovery and mechanistic study of a class of protein arginine methylation inhibitors. J. Med. Chem..

[85] Wang J., Chen L., Sinha S.H., Liang Z., Chai H., Muniyan S., Chou Y.W., Yang C., Yan L., Feng Y., Li K.K., Lin M.F., Jiang H., Zheng Y.G., Luo C. (2012). Pharmacophore-based virtual screening and biological evaluation of small molecule inhibitors for protein arginine methylation. J. Med. Chem..

[86] Horiuchi K.Y., Eason M.M., Ferry J.J., Planck J.L., Walsh C.P., Smith R.F., Howitz K.T., Ma H. (2013). Assay development for histone methyltransferases. Assay Drug Dev. Technol..

[87] Fingerman I.M., Li H.C., Briggs S.D. (2007). A charge-based interaction between histone H4 and Dot1 is required for H3K79 methylation and telomere silencing: Identification of a new trans-histone pathway. Genes Dev..

[88] Min J., Feng Q., Li Z., Zhang Y., Xu R.M. (2003). Structure of the catalytic domain of human DOT1L, a non-SET domain nucleosomal histone methyltransferase. Cell.

[89] Valencia-Sanchez M.I., De Ioannes P., Wang M., Vasilyev N., Chen R., Nudler E., Armache J.P., Armache K.J. (2019). Structural basis of Dot1L stimulation by histone H2B lysine 120 ubiquitination. Mol. Cell.

