# Supplementary material for: The macromolecular complexes of histones affect protein arginine methyltransferase activities
Source: J Biol Chem. 2021 Sep 6;297(4):101123. doi: 10.1016/j.jbc.2021.101123 (PMC8511957; doi:10.1016/j.jbc.2021.101123)

Supporting Information

The Macromolecular Complexes of Histones Affect Protein Arginine Methyltransferase Activities

Melody D. Fulton[†], Mengtong Cao[†], Meng-Chiao Ho[‡], Xinyang Zhao[≠], Y. George Zheng[†]*

[†] Department of Pharmaceutical and Biomedical Sciences, College of Pharmacy, The University of Georgia, Athens, Georgia 30602, United States.
[‡] Institute of Biological Chemistry, Academia Sinica, Nankang, Taipei, Taiwan

[≠] Department of Biochemistry and Molecular Genetics, The University of Alabama at Birmingham, Birmingham, Alabama 35294, United States

Corresponding Author

*Department of Pharmaceutical and Biomedical Sciences, College of Pharmacy, The University of Georgia, Athens, Georgia 30602, United States. Y. G. Zheng, Tel: (706) 542-0277; Fax: (706) 542-5358; Email: yzheng@uga.edu

Figure S1. A whole gel, side-by-side comparison of the main text Figure 2 (panels A and B) with a replicate (panels C and D) radioactive methylation assay with PRMT1. Panels E-H are additional assays of PRMT1 activities. Reactions were performed at 30°C for 30 min with 0.05 μM hPRMT1, 5 μM [^{14}C]SAM, and 1 μM peptide/histone (final concentrations). The negative control contained just enzyme and [^{14}C]SAM. A), C), E), and G) Phosphorscreens were exposed to dried gels for 72 h before scanning the screens with the GE Storm 865 Phosphor Imager at 200 μm resolution. B), D), F) and H) Coomassie blue protein staining of the gels presented in A), C), E), and G), respectively. Lane 10 in all gel images contains the Precision Plus Protein Dual Color Standard (Bio-Rad). Lane 9 was empty in gel images E)-H).

Lane	1	2	3	4	5	6	7	8	9	10
H4 Peptide									+	
H3.3 nuc								+		
H3.3 oct							+			
H4						+				
H3.3					+					
H2B				+						
H2A			+							
PRMT1		+	+	+	+	+	+	+	+	
^{14}C-SAM		+	+	+	+	+	+	+	+	
^{14}C-BSA	+									

Lane	1	2	3	4	5	6	7	8	9	10
H4 Peptide									+	
H3.3 nuc								+		
H3.3 oct							+			
H4						+				
H3.3					+					
H2B				+						
H2A			+							
PRMT1		+	+	+	+	+	+	+	+	
^{14}C-SAM		+	+	+	+	+	+	+	+	
^{14}C-BSA	+									

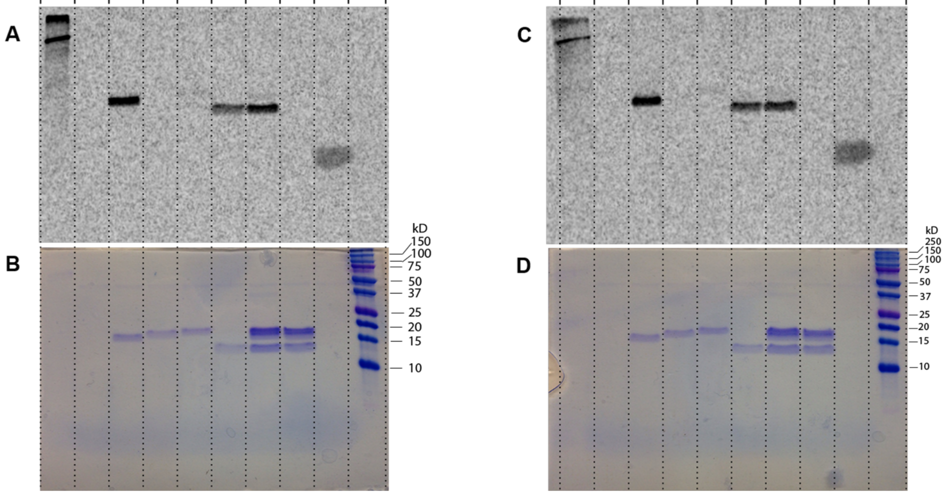

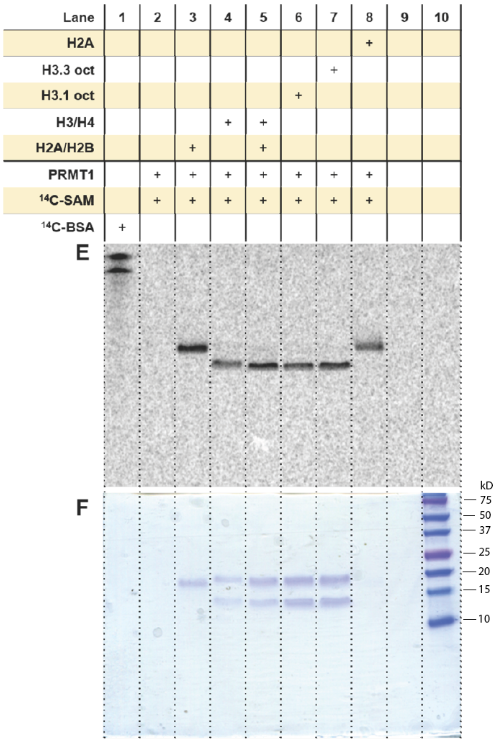
Lane 1 2 3 4 5 6 7 8 9 10
H2A
H3.3 oct
H3.1 oct
H3/H4
H2A/H2B
PRMT1
14C-SAM
14C-BSA
E
F
kD
75
50
37
25
20
15
10

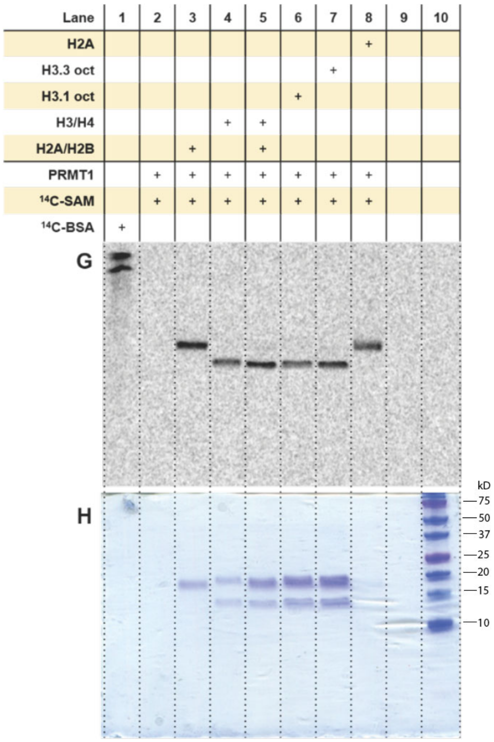
Lane 1 2 3 4 5 6 7 8 9 10
H2A
H3.3 oct
H3.1 oct
H3/H4
H2A/H2B
PRMT1
14C-SAM
14C-BSA
G
H
kD
75
50
37
25
20
15
10

Figure S2. A whole gel, side-by-side comparison of the main text Figures 3A and 3B (panels A and B) with a replicate (panels C and D) radioactive methylation assay with truncated PRMT3. Reactions were performed at 30°C for 30 min with 0.05 μM truncated hPRMT3, 5 μM [^{14}C]SAM, and 1 μM peptide/histone (final concentrations). The negative control contained just enzyme and [^{14}C]SAM. A) and C) Phosphorscreens were exposed to dried gels for 96 h before scanning the screens with the GE Storm 865 Phosphor Imager at 200 μm resolution. B) and D) Coomassie blue protein staining of the gels presented in A) and C), respectively. Lane 10 contains the Precision Plus Protein Dual Color standard (Bio-Rad).

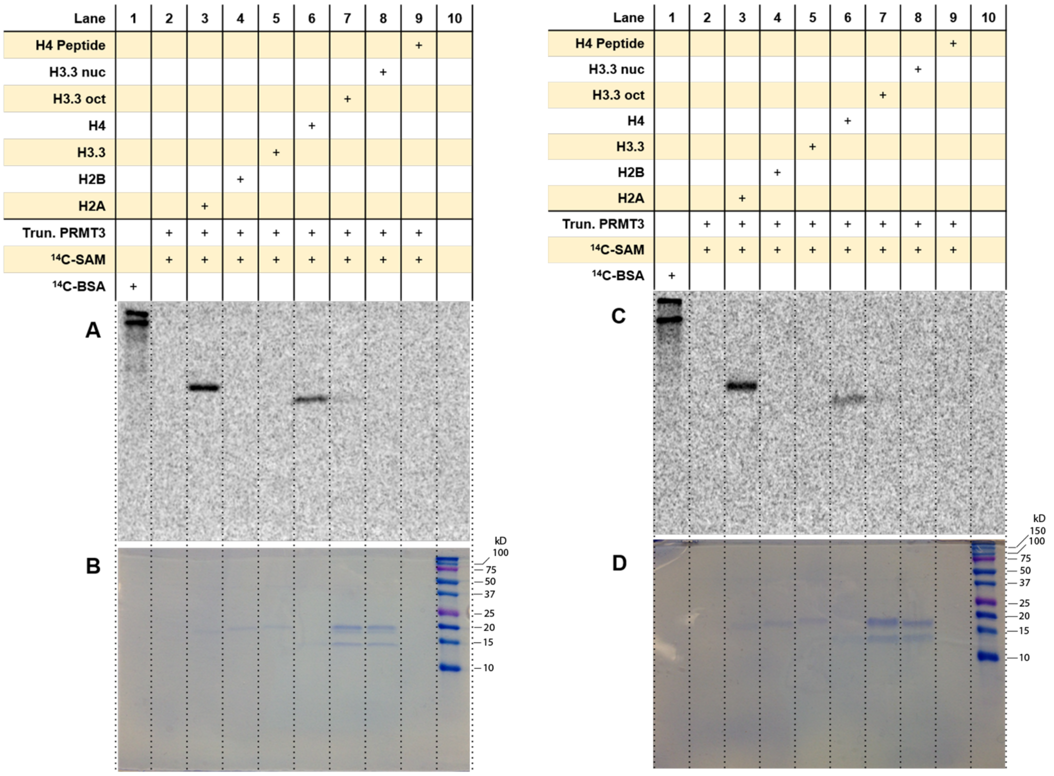

Figure S3. A whole gel, side-by-side comparison of the main text Figures 3C and 3D (panels C and D) with a replicate (panels A and B) radioactive methylation assay with full-length PRMT3. Reactions were performed at 30°C for 30 min with 0.05 μM full-length hPRMT3, 5 μM [^{14}C]SAM, and 1 μM peptide/histone (final concentrations). The negative control contained just enzyme and [^{14}C]SAM. A) and C) Phosphorscreens were exposed to dried gels for 96 h before scanning the screens with the GE Storm 865 Phosphor Imager at 200 μm resolution. B) and D) Coomassie blue protein staining of the gels presented in A) and C), respectively. Lane 10 contains the Precision Plus Protein Dual Color standard (Bio-Rad).

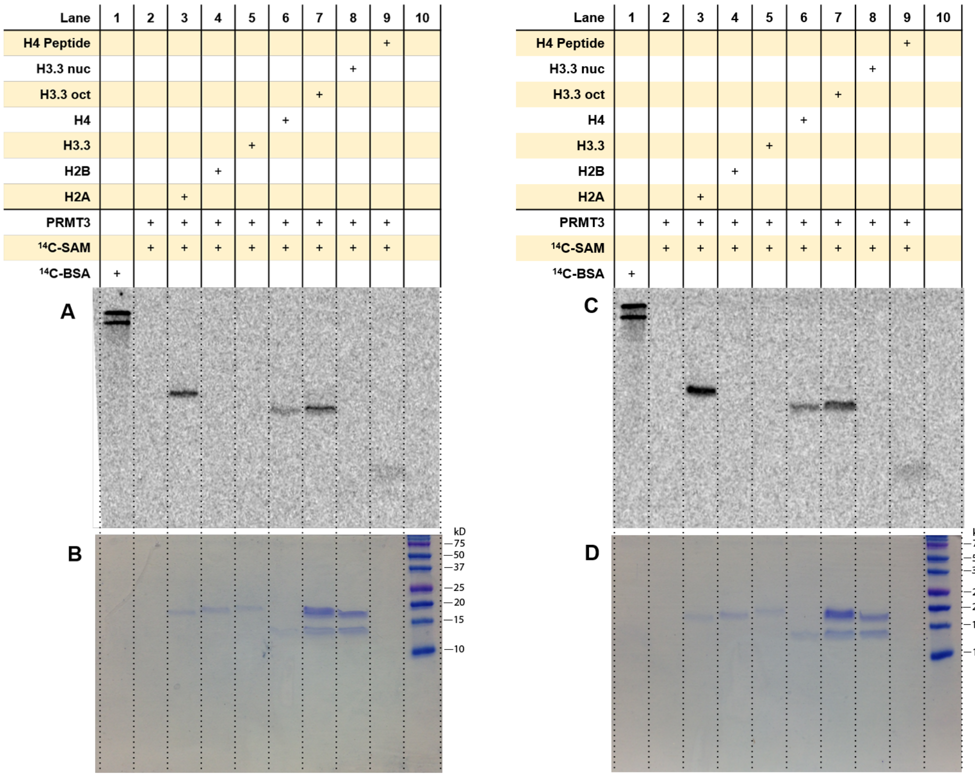

Figure S4. A whole gel, side-by-side comparison of the main text Figure 4 (panels A and B) with a replicate (panels C and D) radioactive methylation assay with hPRMT4 (CARM1). Reactions were performed at 30°C for 1 h with 0.05 μM hPRMT4, 5 μM [^{14}C]SAM, and 1 μM peptide/histone (final concentrations). The negative control contained just enzyme and [^{14}C]SAM. A) and C) Phosphorscreens were exposed to dried gels for 96 h before scanning the screens with the GE Storm 865 Phosphor Imager at 200 μm resolution. B) and D) Coomassie blue protein staining of the gels presented in A) and C), respectively. Lane 10 contains the Precision Plus Protein Dual Color standard (Bio-Rad).

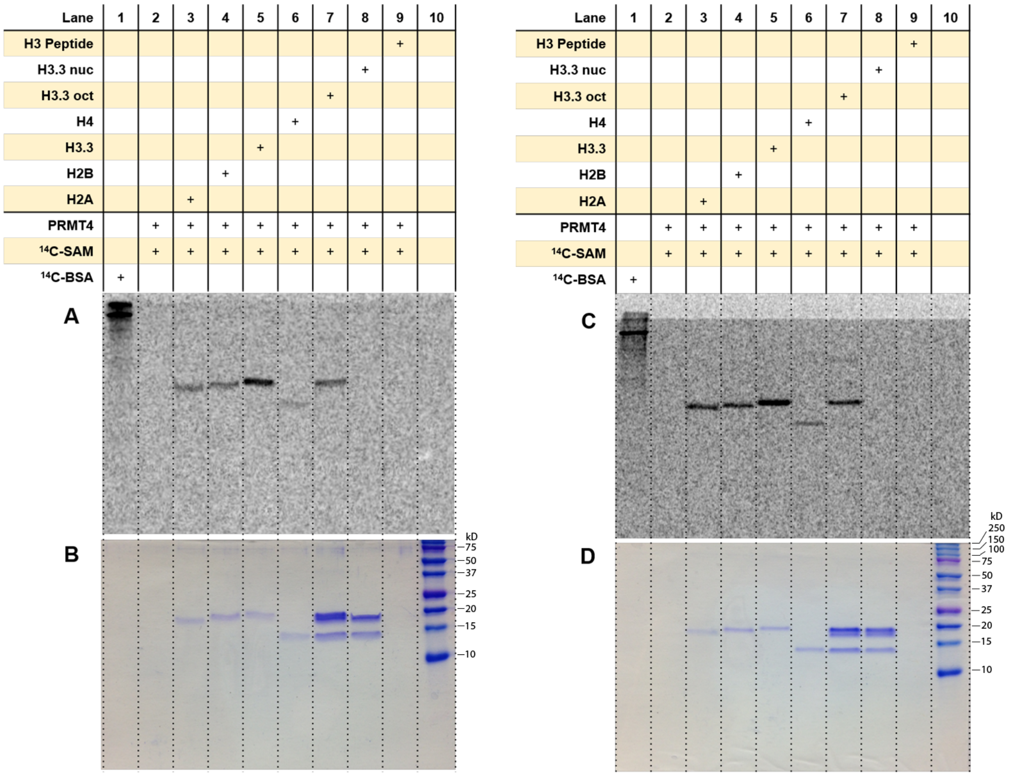

Figure S5. A whole gel, side-by-side comparison of the main text Figures 5A and 5B (panels A and B) with a replicate (panels C and D) radioactive methylation assay with hPRMT5/MEP50. Reactions were performed in duplicate at 30°C for 30 min with 0.05 µM hPRMT5/MEP50, 5 µM [^{14}C]SAM, and 1 µM peptide/histone (final concentrations). The negative control contained just enzyme and [^{14}C]SAM. A) and C) Phosphorscreens were exposed to dried gels for 72 h before scanning the screens with the GE Storm 865 Phosphor Imager at 200 µm resolution. B) and D) Coomassie blue protein staining of the gels presented in A) and C), respectively. Lane 10 contains the Precision Plus Protein Dual Color standard (Bio-Rad).

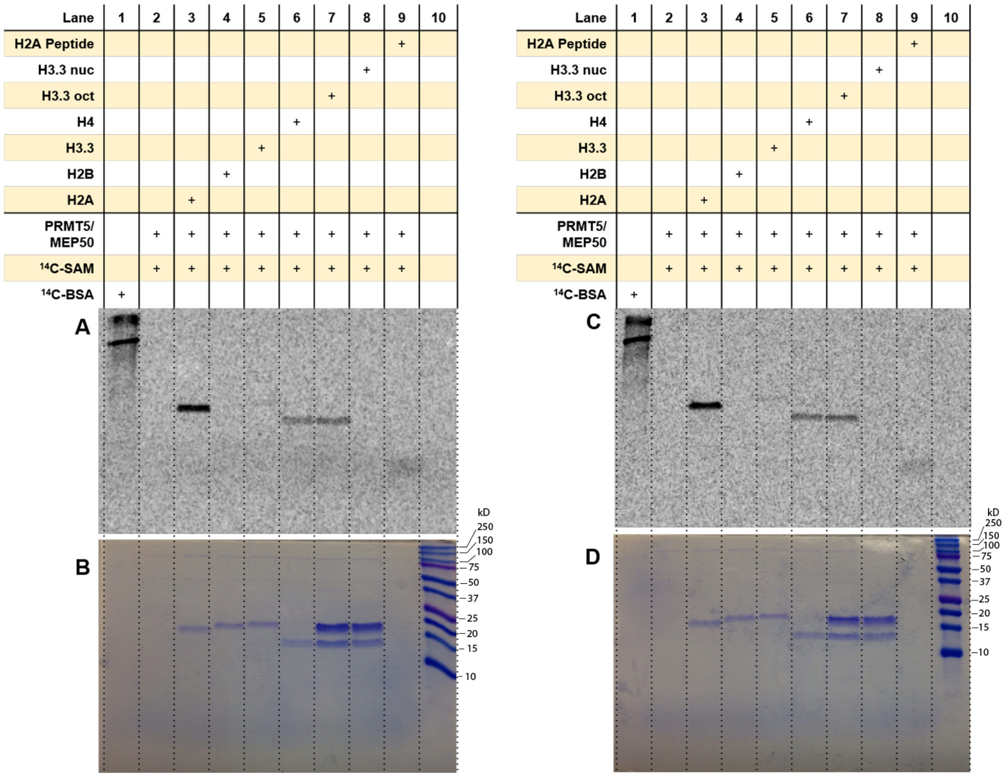

Figure S6. A whole gel, side-by-side comparison of the main text Figures 5C and 5D (panels C and D) with a replicate (panels A and B) radioactive methylation assay with hPRMT5 (without MEP50). Reactions were performed at 30°C for 1 h with 0.2 μM hPRMT5, 5 μM [^{14}C]SAM, and 1 μM peptide/histone (final concentrations). The negative control contained just enzyme and [^{14}C]SAM. A) and C) Phosphorscreens were exposed to dried gels for 96 h before scanning the screens with the GE Storm 865 Phosphor Imager at 200 μm resolution. B) and D) Coomassie blue protein staining of the gels presented in A) and C), respectively. Lane 10 contains the Precision Plus Protein Dual Color standard (Bio-Rad).

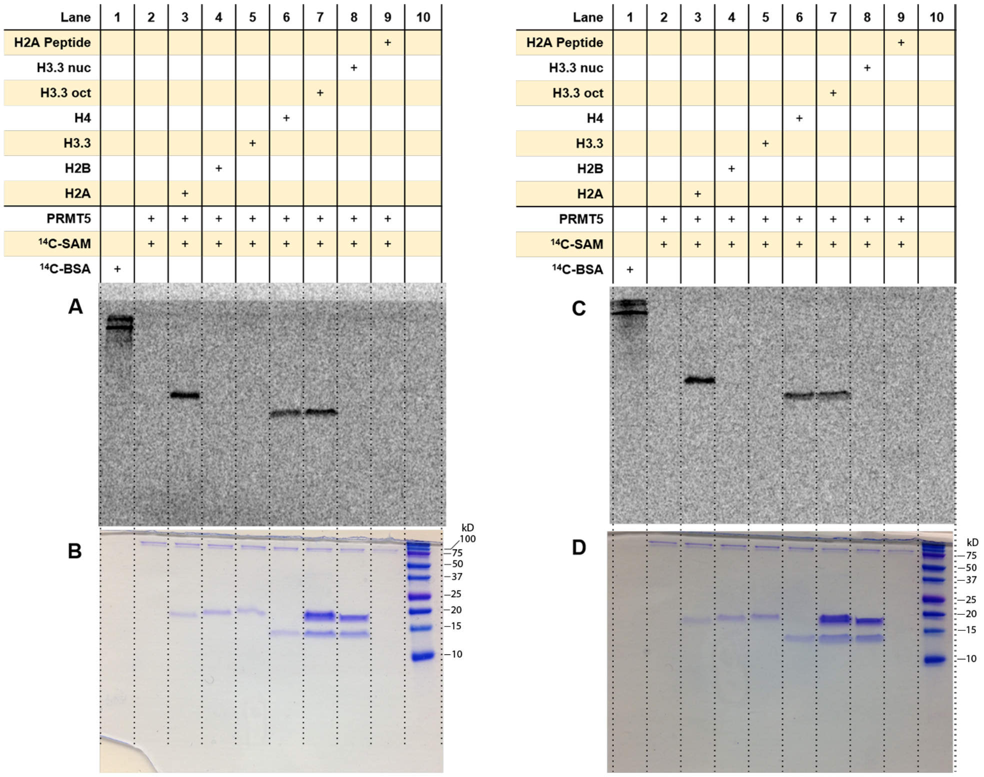

Figure S7. A whole gel, side-by-side comparison of the main text Figure 6 (panels A and B) with a replicate (panels C and D) radioactive methylation assay with hPRMT6. Reactions were performed at 30°C for 3 h with 0.5 μM hPRMT6, 5 μM [^{14}C]SAM, and 1 μM peptide/histone (final concentrations). The negative control contained just enzyme and [^{14}C]SAM. A) and C) Phosphorscreens were exposed to dried gels for 96 h before scanning the screens with the GE Storm 865 Phosphor Imager at 200 μm resolution. B) and D) Coomassie blue protein staining of the gels presented in A) and C), respectively. Lane 10 contains the Precision Plus Protein Dual Color standard (Bio-Rad).

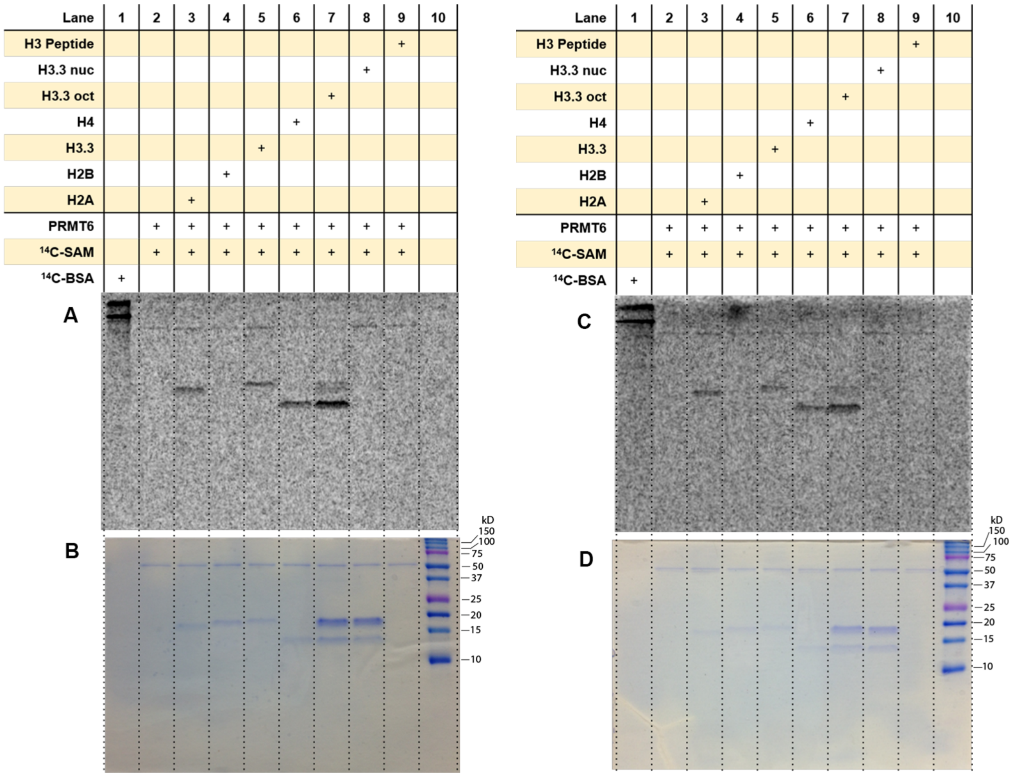

Figure S8. A whole gel, side-by-side comparison of the main text Figure 7 (panels A and B) with a replicate (panels C and D) radioactive methylation assay with hPRMT7. Reactions were performed in duplicate at 30°C for 3 h with 0.5 μM hPRMT7, 5 μM [^{14}C]SAM, and 1 μM peptide/histone (final concentrations). The negative control contained just enzyme and [^{14}C]SAM. A) and C) Phosphorscreens were exposed to dried gels for 96 h before scanning the screens with the GE Storm 865 Phosphor Imager at 200 μm resolution. B) and D) Coomassie blue protein staining of the gels presented in A) and C), respectively. Lane 10 contains the Precision Plus Protein Dual Color standard (Bio-Rad).

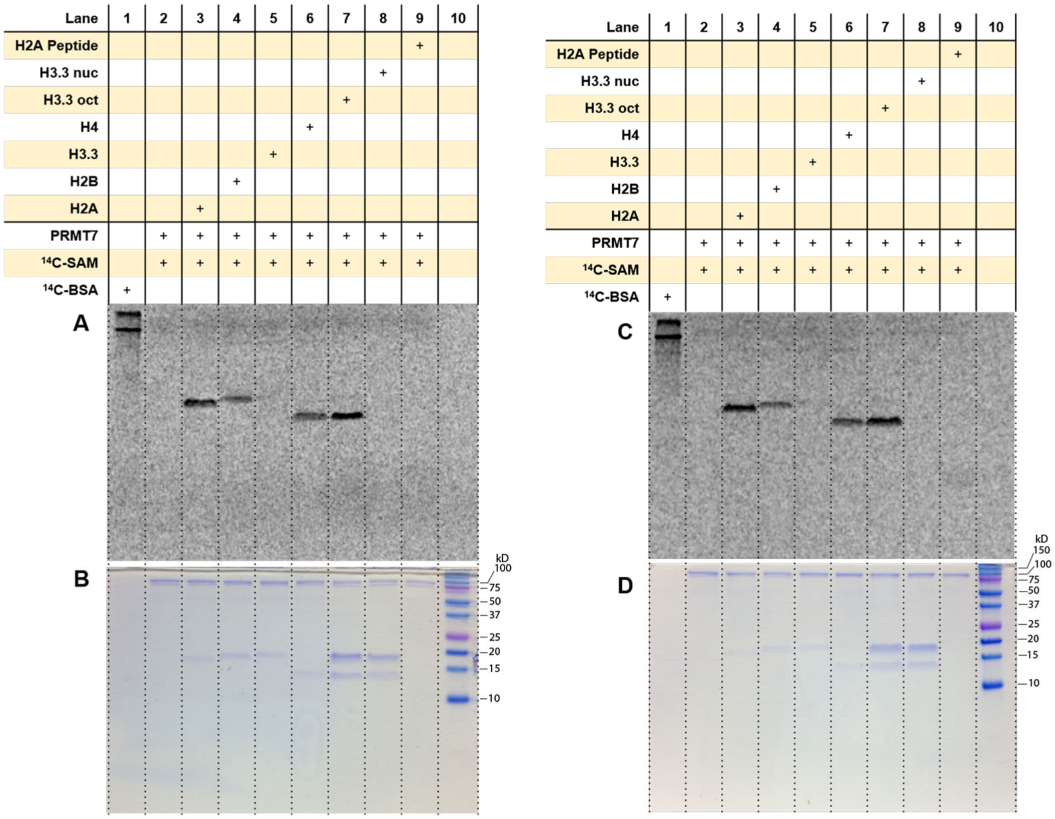

Figure S9. A whole gel, side-by-side comparison of the main text Figure 8 (panels A and B) with a replicate (panels C and D) radioactive methylation assay with hPRMT8. Reactions were performed in duplicate at 30°C for 30 min with 0.05 µM hPRMT8, 5 µM [^{14}C]SAM, and 1 µM peptide/histone (final concentrations). The negative control contained just enzyme and [^{14}C]SAM. A) and C) Phosphorscreens were exposed to dried gels for 96 h before scanning the screens with the GE Storm 865 Phosphor Imager at 200 µm resolution. B) and D) Coomassie blue protein staining of the gels presented in A) and C), respectively. Lane 10 contains the Precision Plus Protein Dual Color standard (Bio-Rad).

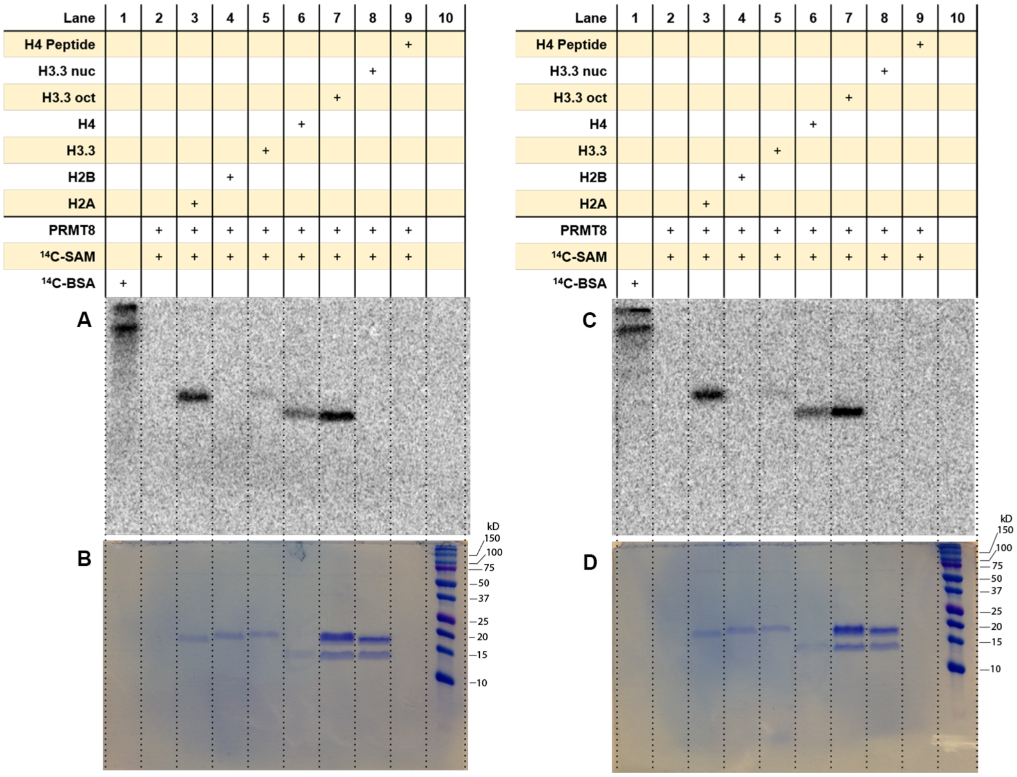

Figure S10. Repeated (gel-based) radioactive methylation assay with hPRMT7. Reactions were performed in duplicate at 30°C for 3 h with 0.5 μM hPRMT7, 5 μM [^{14}C]SAM, and 1 μM peptide/histone (final concentrations). A) Phosphorscreen was exposed to the dried gel for 96 h before scanning the screen with the GE Storm 865 Phosphor Imager at 200 μm resolution. B) Coomassie blue protein staining of the gel presented in A). Lane 10 contains the Precision Plus Protein Dual Color standard (Bio-Rad).

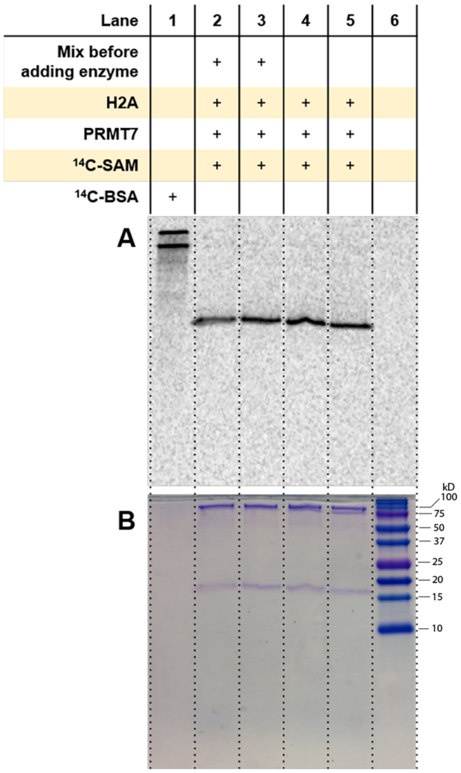

Figure S11. A whole gel, side-by-side comparison of the main text Figure 10 (panels A and B) with a replicate (panels C and D) radioactive methylation assay with PRMT1 and PRMT5/MEP50. Reactions were performed at 30°C for 1 h with 0.05 μM hPRMT1 or 0.05 μM hPRMT5/MEP50, 5 μM [^{14}C]SAM, and 1 μM histone H2A or H3.3 mononucleosome (final concentrations) at low to high NaCl concentrations. In lanes 2 and 6, the final NaCl concentration is approximately 12 mM and 15 mM, respectively. In lanes 3 and 7, the final NaCl concentration is 100 mM. In lanes 4 and 8, the final NaCl concentration is 200 mM. A) and C) Phosphorscreens were exposed to dried gels for 72 h before scanning the screens with the GE Storm 865 Phosphor Imager at 200 μm resolution. B) and D) Coomassie blue protein staining of the gels presented in A) and C), respectively. Lane 10 in both gel images contains the Precision Plus Protein Dual Color Standard (Bio-Rad).

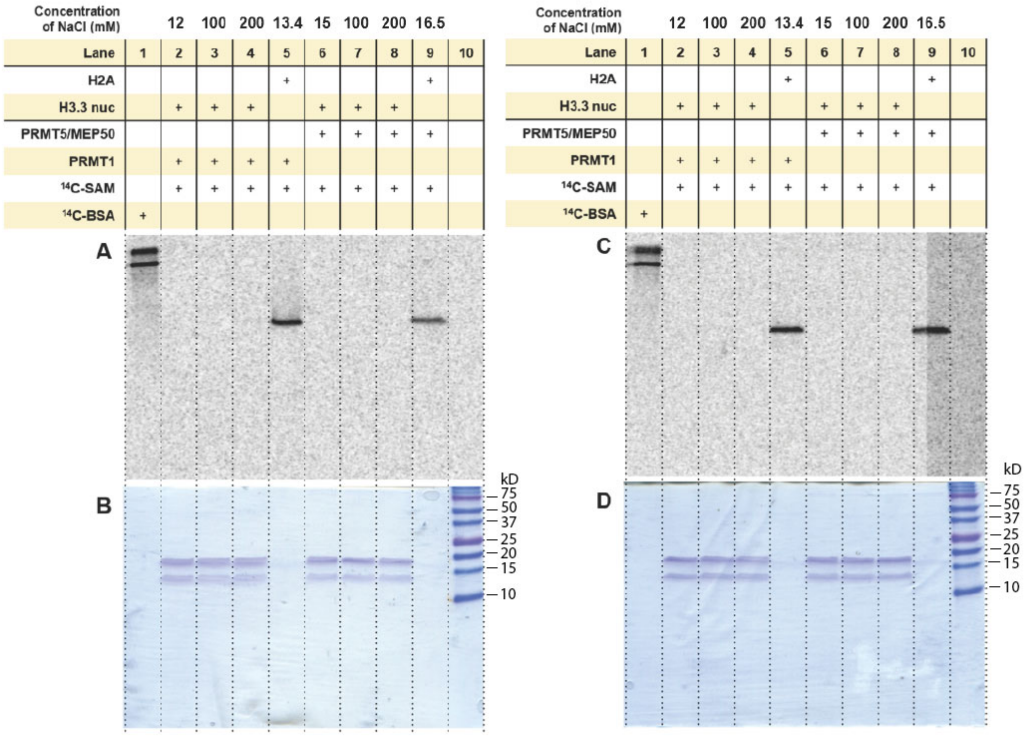

Figure S12. Arginine methylation of histone H4 by PRMT1 and PRMT5/MEP50 in the presence of low to high salt (NaCl) concentrations. Reactions were held for 1 h at 30 °C with 0.05 µM PRMT1 or PRMT5/MEP50, 5 µM [^{14}C]SAM, and 1 µM of substrate. The final NaCl concentration for reactions with PRMT1 and PRMT5/MEP50 were 10 mM, 100 mM, and 200 mM. A) Phosphor image of radiolabeled proteins after 72 h in the dark. B) Coomassie blue protein staining. [^{14}C]BSA and a protein ladder were used in lanes 1 and 8 as a radiolabeled protein marker and a protein kDa reference, respectively.

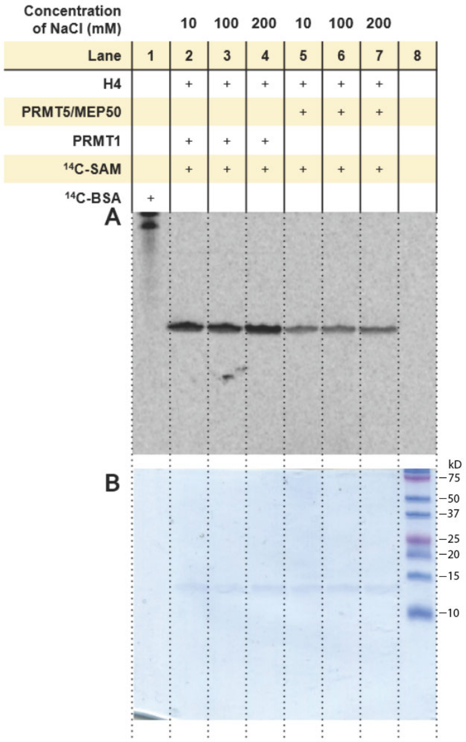

Figure S13. Comassie blue staining of the Native PAGE gel. 6 μL of mononucleosome, 6 μL of recombinant histone H4 and 18 μL of PRMT1 were kept on ice and diluted by reaction buffer (50 mM HEPES pH 8, 10 mM NaCl, 0.5 mM EDTA, 0.5 mM DTT) to make 30 μL of reaction system. The mixtures were incubated for 2 h at 30°C. The final concentrations of each component in the reaction system were 1 μM of mononucleosome, 1 μM of recombinant histone H4 and 1 μM of PRMT1. After 2 h incubation, 6 μL of 5x sample buffer (1 M tris, 1% solution of bromophenol blue, 50% glycerol) was added. 30 μL of reaction sample was loaded into each well of a Native PAGE gel (10% separating gel, 4% stacking gel). Proteins were resolved with 100V for 70 min in the electrophoresis buffer (0.192M glycine, 0.025M tris). Gel was soaked in Coomassie staining solution (10% acetic acid, 45% methanol, 3% glycerol, 41.5% water, 0.5% Coomassie Blue) for 20 min and then soaked in destaining solution (10% acetic acid, 45% methanol, 3% glycerol, 42% water) for 40 min.

Lane	1	2	3	4	5
PRMT1	+			+	+
H4		+		+	
mononucl eosome			+		+

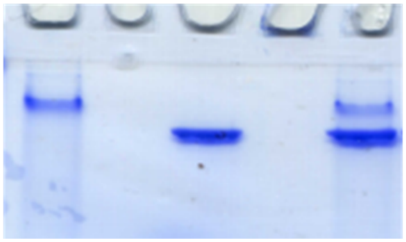

Figure S14. A proposed model on PRMT substrate recognition primed by electrostatic attraction. We suggest that PRMT-substrate interaction is significantly primed by the electrostatic charge-charge attraction between the acidic surface of PRMTs and the alkaline surface of their substrates, and only under this interactive context, specific bindings come into play to fine tune individual arginine site specificity.

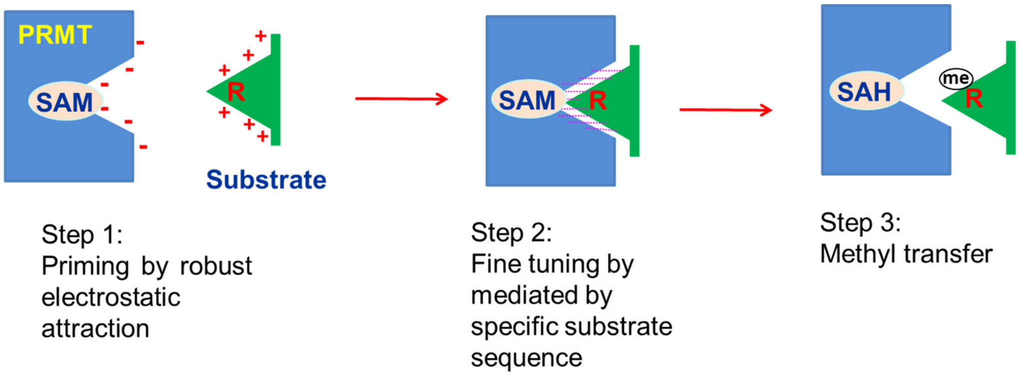

Figure S15. ^{1}H NMR spectra of the histone Ac-H4(1-20) peptide. 1 mM of 2,2-dimethyl-2-silapentane-5-sulfonate sodium salt (DSS) was used as an internal standard.

$$C_x = \frac{I_x}{I_{cal}} \times \frac{N_{cal}}{N_x} \times C_{cal}$$

Calculation of Adjustment Factor
Expected concentration based on weight = 0.901 mM
^{1}H NMR based concentration = 0.77 mM
Adjustment factor = 0.77 mM/0.901 mM ≅ **0.85**

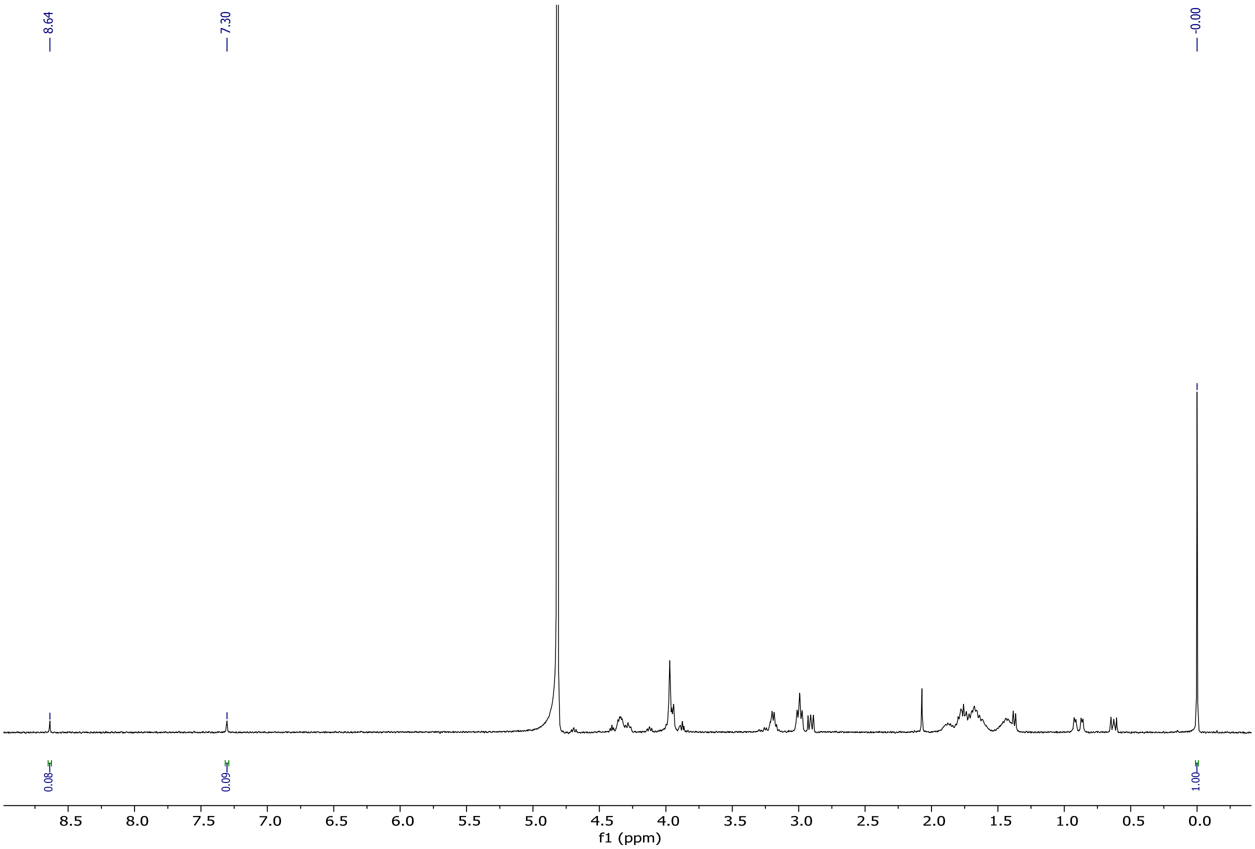

Supplement: Supplemental Figures S1–S15 [file mmc1.pdf]
